# Foreign shareholder, overseas sale and corporate profit margin

**DOI:** 10.1371/journal.pone.0296021

**Published:** 2024-02-05

**Authors:** Caihong Wen

**Affiliations:** School of Accounting, Southwestern University of Finance and Economics, Chengdu, Sichuan, China; Lingnan University, The University of HongKong, HONG KONG

## Abstract

China is actively promoting the development of a robust trading nation. In this context, utilizing data from China’s A-share listed companies spanning from 2003 to 2021, this study investigates the impact of foreign shareholders on enterprises in a scenario where overseas sales reduce the profit margin of Chinese firms. The findings reveal that overseas sales do indeed decrease the profit margin of Chinese enterprises; however, foreign shareholders mitigate this negative effect and various robustness tests support this conclusion. Mechanism analysis confirms that foreign shareholders primarily enhance enterprise productivity through improved production technology spillover effects, thereby alleviating the adverse impact of overseas sales on Chinese firms’ profit margins. Heterogeneity analysis demonstrates that both longer holding periods for foreign shareholders and multiple foreign shareholders significantly alleviate the negative influence of overseas sales on Chinese firms’ profit margins. Moreover, there is significant heterogeneity in how foreign shareholders alleviate these detrimental consequences based on property rights nature, institutional environment, overseas related party transactions and subsidiaries, as well as industry attributes. These findings have important reference value for China’s efforts towards becoming a strong trading nation and can contribute to enhancing trade capacity in other countries.

## 1. Introduction

**Fig 1:**
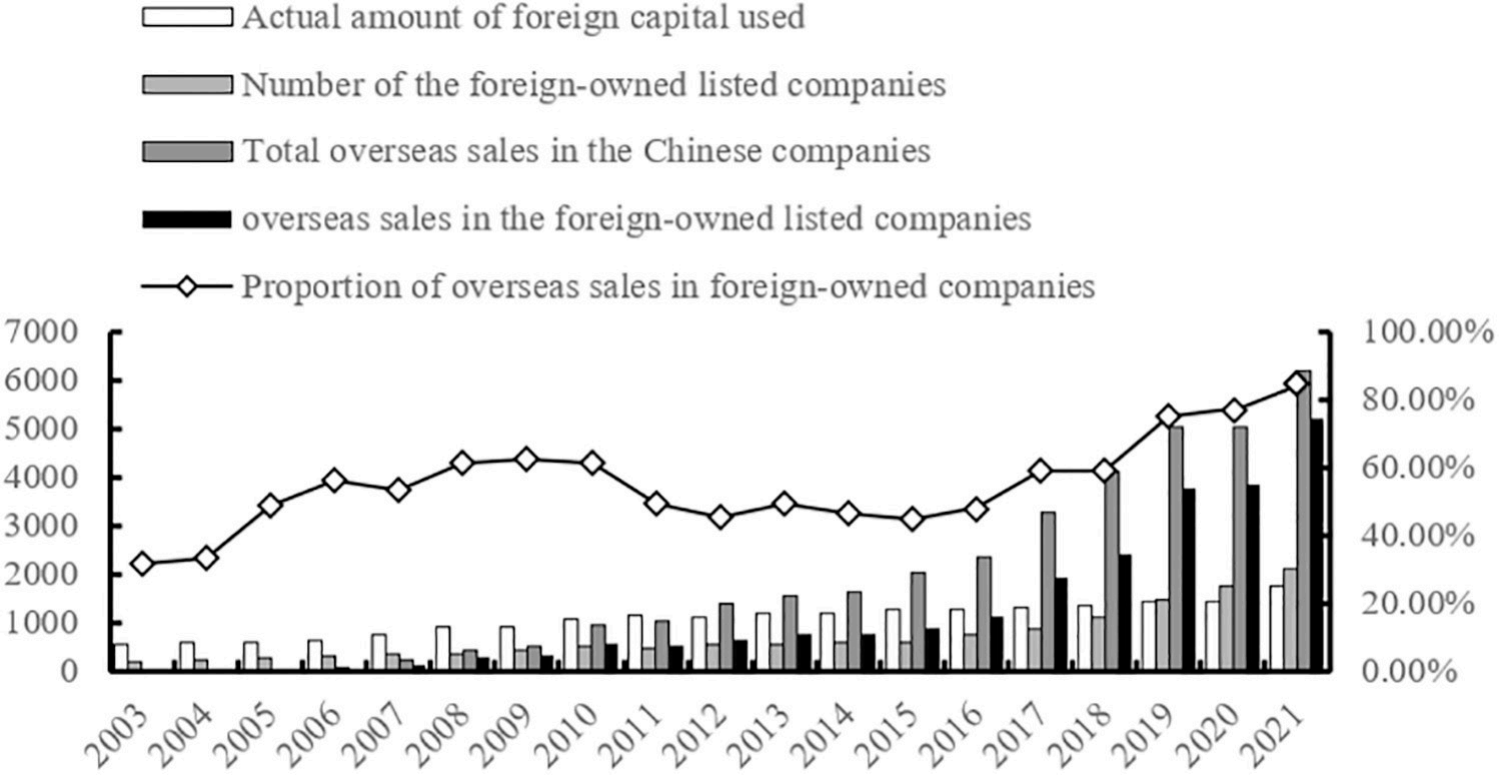
Foreign investment and overseas sale in China.

Since joining the WTO in 2001, China has continuously strengthened its position in global trade, ultimately becoming the world’s largest trading nation. The Chinese Ministry of Commerce reports that foreign direct investment inflows into China have steadily increased each year and reached an impressive $173.4 billion in 2021 with a growth rate of 20.16%. Additionally, there has been a significant rise in the number of foreign-owned companies operating within China. According to data from the Chinese General Administration of Customs, China’s export scale has experienced rapid expansion since joining the WTO and reached a total export volume of ¥21,690.8 billion yuan in 2021 with a growth rate of 20.99%. At a micro level analysis reveals that as exports surge, overseas sales by enterprises have also witnessed substantial growth totaling ¥68,322.2 billion yuan in 2021 with an impressive increase of 36.11%. It is worth noting that more than four-fifths (84%) of these overseas sales are attributed to foreign-owned enterprises (Fig 1), indicating their crucial role in promoting oversea sales for Chinese firms through introducing foreign shareholders into their operations.

However, extensive research on the performance of Chinese export enterprises consistently indicates a productivity gap between export and non-export firms, revealing the existence of an "export-productivity paradox" among Chinese companies [1,2]. Consequently, as overseas sales by Chinese enterprises continue to grow, their productivity and profit margins tend to decline [3]. This underscores China’s current challenge of being "big but not strong" in international trade. While previous literature extensively examines the influence of foreign shareholders on overseas sales and their quality for Chinese enterprises [4–6], it has yet to explore their impact on the profit margin associated with such sales. Therefore, this study aims to investigate whether foreign shareholders affect the relationship between overseas sales and corporate profit margin in order to provide insights into how foreign shareholders may operate under conditions where higher overseas sales correspond with lower profit margins for Chinese firms.

Understanding the impact of corporate participants, such as shareholders and management, on enterprises and the socio-economy as a whole is imperative. Shareholders and management play pivotal roles in making internal decisions within enterprises, with their characteristics influencing behavioral choices that subsequently affect efficiency at both enterprise and socio-economic levels. An et al. [7] conducted a transnational study involving 93,697 companies to examine the effects of war on countries and corporations. The findings revealed an increase in directors with military backgrounds on corporate boards in war or later; however, these directors were associated with diminished corporate performance measured by Tobin’s Q and return on assets (ROA). Shi et al. [8] investigated the influence of controlling shareholders’ financial background on corporate financialization, finding significant promotion of this phenomenon due to such backgrounds. Agarwal and Chaudhry [9] examined non-financial listed companies in India to explore how foreign shareholders impact corporate investment behavior; they discovered that having foreign shareholders led to decreased investments by corporations. Considering China’s unique institutional context, we aim to explore the impact of shareholder foreignness on the relationship between overseas sales and corporate profitability in order to provide empirical evidence for studying the influence of corporate participants on firms.

This study aims to analyze the influence of foreign investment on the correlation between overseas sales and profits of Chinese enterprises, as well as investigate the underlying mechanisms involved. Specifically, we examine a sample of China’s A-share listed companies from 2003 to 2021 to explore how foreign shareholders impact the phenomenon where overseas sales lead to reduced corporate profit margins. To ensure robustness, various methods are employed for conducting rigorous tests. Additionally, we delve into the production technology spillover effect and information spillover effect as potential mechanisms through which foreign investment affects the relationship between overseas sales and profit margins of Chinese enterprises. Furthermore, we analyze heterogeneity among foreign shareholders based on their duration of shareholding and number. Finally, this paper explores how factors such as property rights nature, institutional environment, overseas related party transactions, overseas subsidiaries, and industry attributes contribute to understanding the impact of foreign shareholders on reducing corporate profit margins resulting from overseas sales.

The spillover effect and profit-seeking behavior of foreign shareholders indicate that they can have a dual impact on the phenomenon of Chinese enterprises’ declining corporate profit margins due to overseas sales. Foreign shareholders can directly leverage their knowledge of overseas markets to reduce the expenses associated with preliminary market research for Chinese enterprises’ overseas sales. Additionally, the established overseas sales channels of foreign shareholders can help mitigate risks and expenses related to Chinese enterprises’ international expansion, thereby alleviating the negative impact on corporate profit margins. Moreover, foreign investment brings about technology spillovers through demonstration effects and personnel flow effects [10], which enhance enterprise productivity, indirectly improve the quality of products sold abroad, and reduce costs associated with international sales. Based on these spillover effects, foreign shareholders are able to mitigate the adverse consequences arising from Chinese enterprises’ overseas sales.

However, the profit-seeking behavior of foreign shareholders in pursuit of their own interests may potentially amplify the adverse impact of overseas sales on the profit margin of Chinese enterprises. Motivated by labor acquisition, risk aversion, and financial speculation, foreign shareholders might perceive overseas sales as a means to obtain personal benefits. For instance, they could exploit information asymmetry related to overseas sales for insider trading and other unethical practices that harm the enterprise’s interests. Consequently, these actions by foreign shareholders would further exacerbate the negative consequences of overseas sales on enterprises’ profit margins.

The findings of this study demonstrate that Chinese enterprises experience a decrease in profit margin as their overseas sales increase, indicating that overseas sales have an adverse effect on the profitability of these enterprises. However, foreign shareholders play a significant role in mitigating the negative impact of overseas sales on Chinese enterprises’ profit margins. Various robust methods, including dynamic adjustment of foreign shareholders, propensity score matching method, Heckman two-stage regression analysis, consistently support the aforementioned conclusion. The results from the analysis of impact mechanisms reveal that foreign shareholders alleviate the negative impact of overseas sales on profit margins not through information spillover effects but primarily by facilitating production technology spillover effects to enhance productivity for Chinese enterprises. Heterogeneity analysis further indicates that longer holding periods and multiple foreign shareholders significantly contribute to reducing the detrimental effect of overseas sales on Chinese enterprises’ profit margins. Moreover, additional analyses suggest that this phenomenon is more pronounced in non-state-owned enterprises, particularly private ones. Furthermore, China’s institutional environment exhibits heterogeneous impacts on how foreign shareholders mitigate the negative consequences of overseas sales on corporate profit margins; notably, such mitigation is more prominent in regions with well-established legal systems. Simultaneously, it becomes evident that foreign shareholders are better able to counteract the adverse impact of overseas sales on corporate profit margins when there are no overseas related party transactions or fewer overseas subsidiaries. Additionally, the impact of foreign shareholders mitigating the negative effects of overseas sales on corporate profit margins varies depending on industry attributes. This effect is more pronounced in industries that are non-polluting and have lower capital intensity.

Compared to the existing literature, this paper makes several significant contributions. Firstly, while a substantial body of literature has examined the impact of corporate governance participants on corporate performance [7], this study investigates the influence of shareholder characteristics on enterprises within the institutional context of China as a developing country. This empirical evidence contributes to expanding the understanding of such literature in developing countries. Secondly, previous research has demonstrated that foreign shareholders facilitate overseas sales for companies [11]. Building upon these findings, this paper further explores the economic consequences associated with foreign shareholders promoting overseas sales for enterprises, thereby extending existing knowledge in this area. Thirdly, against the backdrop of China’s active pursuit to become a strong trading nation, this study examines how foreign investment affects the relationship between overseas sales and profit margins for Chinese enterprises. The results reveal that foreign investment mitigates the negative impact of overseas sales on profit margins for Chinese enterprises, suggesting that it not only facilitates international expansion but also enhances profitability. Consequently, attracting foreign investment can contribute to China’s ambition to build a robust trading nation while providing empirical evidence relevant to other countries’ trade capacity development efforts—particularly those in developing stages. Lastly, the heterogeneity analysis of foreign shareholders in this study reveals that long-term shareholding and multiple foreign shareholders can mitigate the adverse impact of overseas sales on Chinese enterprises’ profit margin. Therefore, it is advisable to encourage foreign investors to engage in long-term investments and strategically structure the ownership composition of foreign shareholders when attracting foreign investment. This finding offers valuable insights for other developing nations seeking to optimize their ownership design while attracting foreign investment. Additionally, our further analysis demonstrates the need for enhanced supervision over overseas related party transactions and subsidiaries within foreign-owned enterprises as a means to safeguard the interests of Chinese enterprises and shareholders. Such measures hold significant practical implications in curbing profit transfer through overseas sales by foreign investors. These conclusions provide empirical evidence from China that can guide other developing countries in harnessing positive spillover effects from their own foreign shareholder arrangements.

The paper is structured as follows: Section two presents a comprehensive literature review and outlines the research hypothesis; Section three provides an overview of the research design; Section four presents the main econometric results along with robustness tests; Section five offers an in-depth mechanism analysis; Section six conducts heterogeneity analysis, and finally, a concise summary of the entire paper is provided.

## 2. Literature review and research hypothesis

As the world’s largest and most influential developing country, China has been steadily enhancing its international status and influence in global affairs. Consequently, examining the financial and economic implications of various factors within the Chinese context can provide valuable insights for the economic development of other countries, particularly those that are still in the process of development. For instance, Chen et al. [12] discussed business founders influenced by Confucian culture tend to choose family or related individuals as successors. Unlike developed countries where such succession often negatively impacts corporate performance, in China’s capital market, family or related successors have a positive effect on corporate performance. Tsafack and Guo [13] explored foreign investment introduction in China and demonstrated an inverted U-shaped relationship between the shareholding ratio of foreign shareholders and corporate performance. Additionally, Bao and Huang [14], as well as Li et al. [15], examined the impact of the COVID-19 outbreak on rural households and non-bank financial institutions (specifically fintech lending institutions) in China. These examples highlight that due to China’s unique institutional background, it is crucial to explore various factors’ financial and economic consequences.

Given the unique institutional context in China, our study aims to examine the influence of foreign capital characteristics on enterprises and even the economy by analyzing how foreign shareholders impact the relationship between overseas sales and corporate profit margins of Chinese firms. Consequently, our research primarily focuses on conducting a comprehensive literature review from the perspectives of overseas sales and foreign shareholders.

### 2.1 Overseas sales

#### 2.1.1 Productivity enhances export of corporations

The theory of heterogeneous corporate trade posits that the primary characteristic of corporate heterogeneity lies in productivity disparities, which subsequently determine the extent of their export activities. This theory is substantiated by a plethora of studies consistently demonstrating a positive correlation between export engagement and elevated levels of productivity. The underlying rationale for this correlation can be attributed to two fundamental effects: the self-selection effect and the export learning effect [16,17].

The productivity of companies that choose to export their products is significantly influenced by the "self-selection" effect, which arises from the higher costs involved in entering foreign markets compared to domestic ones. As a result, only highly productive companies are able to afford the self-selection process and venture into international markets [18,19]. These companies are often characterized by their larger size, stronger establishment, and possession of the necessary resources for effectively navigating the complexities of global trade.

The productivity of exporting companies can be significantly enhanced by the "export learning" effect. When companies expand into foreign markets, they have the opportunity to achieve economies of scale in larger international markets. This leads to increased production and improved corporate performance through lower unit costs and higher profit margins. Additionally, technology assimilation from other countries allows exporting companies to enhance their own technological capabilities. This knowledge transfer process, known as technology transfer, can result in substantial productivity improvements [20,21]. By observing and adopting best practices from other countries, companies can optimize their production processes, leading to improved efficiency and competitiveness.

In summary, the increased productivity of companies that choose to export their products can be attributed to both the phenomenon of "self-selection" and the benefits derived from learning through exporting. The former ensures that only the most efficient companies enter foreign markets, while the latter facilitates knowledge acquisition and adaptation, leading to advancements in technology and production processes. Together, these factors create a positive cycle that motivates companies to continue exporting and continuously enhance their productivity.

#### 2.1.2 The “export-productivity paradox” in China

However, numerous studies have discovered that the productivity of Chinese companies involved in exportation is notably lower than that of their domestically focused counterparts. This phenomenon, referred to as the "export-productivity paradox" in the literature, suggests that as Chinese companies increase their export intensity, their productivity and profit margins tend to decrease [22–24]. In essence, Su and Hong [3] discovered that Chinese companies engaged in export-related activities tend to exhibit lower profit margins compared to their domestic counterparts. This issue has garnered significant attention from researchers, who have conducted in-depth analyses to unearth the underlying reasons for this apparent contradiction. The findings of these studies indicate that several factors contribute to the low productivity of Chinese companies engaged in exportation.

Firstly, labor-intensive products have been identified as a crucial factor. Lu [25] examined the existence of paradoxes based on the capital-labor ratio across different enterprises, revealing a more pronounced manifestation in labor-intensive industries while being absent in capital-intensive sectors. Secondly, the processing trade, a trading model where imported raw materials are processed and exported without substantial value-added, has been pinpointed as another potential reason for the low productivity of Chinese export companies. Dai et al. [24] find that the prevalence of processing trade in China often leads to a more labor-intensive production process, thereby reducing productivity levels. Thirdly, market segmentation and local protection have also been cited as potential causes for the export-productivity paradox. Sheng [26], utilizing micro-data from Chinese industrial enterprises spanning 1998 to 2006 and employing the Heckman two-stage selection model, investigated the "productivity paradox" of enterprise exports. The findings indicate that local administrative monopolies in China impose numerous restrictions and policy guidance on foreign enterprises, distorting their export behavior and serving as significant factors contributing to the emergence of the "productivity paradox". Yang and He [23] found that local protection exists in the Chinese market with high profits associated with it; however, only enterprises with high productivity can share and obtain these protected profits while those with low productivity enter into export markets resulting in an "export-productivity paradox".

Many studies have consistently demonstrated that Chinese corporations engaged in export activities exhibit lower productivity levels compared to their domestic counterparts, thus giving rise to the "export-productivity paradox" phenomenon [22–24]. Consequently, as Chinese corporations increase their export intensity, it is expected that both productivity and profit margins will decline [3]. Furthermore, previous research investigating the underlying causes for this lower productivity among Chinese exporting firms has identified factors such as reliance on labor-intensive products, involvement in processing trade, market segmentation, and local protectionism [22,25,26].

### 2.2 Foreign shareholder

Numerous studies have extensively examined the economic implications of foreign shareholders, yielding a substantial body of research findings. For instance, these investigations have explored the influence of foreign shareholders on various aspects such as cash dividends [27], social responsibility performance [28,29], productivity [30,31], and profit margins [32,33]. However, a consistent evaluation regarding the impact of foreign shareholders has yet to be established.

Several studies suggest that foreign shareholders have a positive spillover effect on local corporations [34–36]. In comparison to domestic shareholders, foreign shareholders, being owners of advanced technology and management concepts, can enhance the technological and managerial efficiency of local corporations through technology transfer and demonstration effects. Fosfuri et al. [10] find that technological spillovers from foreign direct investment occur when these workers are subsequently employed by local firms. Therefore, Arnold and Javorcik [37] propose that foreign ownership leads to substantial productivity improvements within the acquired plants. These enhancements become evident during the year of acquisition and persist in subsequent periods. Lin et al. [30] discover that Hong Kong, Macao, and Taiwan (HMT) invested firms generate negative horizontal spillovers while non-HMT foreign invested firms (mostly from OECD countries) tend to bring about positive horizontal spillovers in China. Cao and Chen [38], using multi-year enterprise panel data from 2000 to 2007, construct an analysis on the influence of foreign direct investment along with other factors on the quality of enterprises’ export products. The conclusion drawn is that foreign direct investment significantly promotes the quality of export products.

On the contrary, certain studies suggest that the entry of foreign shareholders on a large scale may have a detrimental spillover effect on local corporations. Konings [39] empirically examines the impact of foreign direct investment (FDI) on the productivity performance of domestic firms in three emerging economies in Central and Eastern Europe, namely Bulgaria, Romania, and Poland. The findings indicate an absence of significant evidence supporting positive spillover effects on domestic firms across these three countries. Furthermore, negative spillovers are observed for domestic firms in Bulgaria and Romania, while no spillover is observed for domestic firms in Poland. Mao and Xu [40] analyze the impact of foreign entry on local firms’ domestic value-added ratio (DVAR) using Chinese micro-data and find that foreign entry reduces local firms’ export DVAR through horizontal spillover effects. Consequently, the presence of foreign shareholders intensifies competition in the local market and enables them to capture a substantial share from domestic corporations. Chang and Xu [41] highlight the relative magnitude of spillover effects and competition effects. Their findings indicate that while the entry of foreign firms generally benefits domestic companies nationwide, it adversely affects survival rates of local firms in regional markets. Luo and Ge [42] conduct a comprehensive assessment regarding the impact of foreign shareholders on China’s independent R&D by utilizing R&D expenditure data from manufacturing enterprises above designated size spanning from 2005 to 2007. The findings demonstrate that multinational companies diminish autonomous research and development within China’s manufacturing industry as evidenced by reduced research and development propensity as well as intensity.

After conducting a comprehensive analysis of the literature, it becomes apparent that there is a clear inverse correlation between overseas sales and corporate profit margin in Chinese enterprises. Essentially, as the volume of overseas sales increases, the company’s profit margin tends to decrease. This indicates that increased overseas sales have a negative impact on corporate profitability. Furthermore, previous research on the influence of foreign shareholders has not reached a consensus. While some studies highlight their positive influence, others focus on potential negative effects. However, most of these studies fail to consider the potential impact of foreign shareholders on the relationship between overseas sales and corporate profit margin. Therefore, overseas sales reduce corporate profits in Chinese companies can serve as an invaluable case study for exploring economic implications associated with foreign shareholder involvement. This research provides valuable empirical evidence regarding economic consequences resulting from foreign shareholders’ presence in firms operating within developing countries like China. It enhances our understanding of intricate interactions among foreign shareholders, overseas sales, and corporate profit margins while offering insights into effective management strategies for achieving long-term success in global markets.

### 2.3 Research hypothesis

#### 2.3.1 Positive spillover effect of foreign shareholder

Foreign shareholders can exert a positive spillover effect on Chinese corporations through the dissemination of information and technology, thereby mitigating the adverse impact of overseas sales on profit margins.

Based on the information spillover effect, entering overseas markets from domestic markets requires a substantial increase in costs for corporations. These costs include marketing expenses for establishing overseas distribution channels and service networks, management expenses for overseas sales activities, and transportation expenses during the process of overseas sales [16]. Consequently, compared to domestic sales, the expenses associated with overseas sales are higher. As a result, the profit margin decreases as the volume of overseas sales increases [3]. However, foreign shareholders not only bring capital to Chinese corporations but also provide valuable insights into international markets. Therefore, foreign shareholders can leverage their knowledge of overseas market conditions such as competition levels, product distribution patterns abroad, and consumer preferences in order to reduce early market research costs. Additionally, once a foreign shareholder becomes involved with a Chinese corporation, that corporation gains access to other companies owned by the same foreign shareholder. This allows Chinese corporations to utilize existing sales channels within these affiliated companies and thereby mitigate risks and reduce expenses associated with selling products internationally [43]. By directly reducing uncertainty and overheads related to overseas sales operations, foreign shareholders effectively alleviate the negative impact on profit margins caused by engaging in international trade.

Based on the technology spillover effect, Chinese corporations mainly sell labor-intensive and low-technology products, low-value-added products, and raw material processed products in overseas markets. This leads to limited productivity improvement even after overseas sales due to the core technology being held by developed countries’ corporations. However, foreign shareholders not only bring capital but also advanced technology and management ideas that can promote productivity through demonstration effects and personnel flow effects. Therefore, foreign shareholders indirectly improve product quality sold overseas while reducing costs by improving productivity. As a result, they mitigate the negative impact of overseas sales on corporate profit margins.

In conclusion, foreign shareholders have the potential to mitigate the adverse impact of overseas sales on corporate profit margins by directly reducing expenses associated with such sales and indirectly enhancing corporate productivity. Therefore, we propose research Hypothesis 1.

H1: Foreign shareholder mitigates the negative impact of overseas sales on corporate profit margins.

#### 2. 3.2 Beneficial effect of foreign shareholder

As the world’s most populous country, China possesses abundant cheap labor resources and vast market potential. Meanwhile, as a developing nation, China has temporarily relaxed its control over energy development and industrial pollution emissions. Acquiring labor resources, capturing market share, and consuming energy while emitting pollutants have become the primary motivations for foreign investors entering China. When foreign shareholders enter the Chinese corporate sector, they can leverage China’s factor endowment and utilize Chinese corporations as export platforms or to cater to the domestic market. Consequently, Chinese corporations transform into processing factories for foreign shareholders, leading to a lower productivity of foreign corporations engaged in exports compared to those not involved in exporting [22]. The motivations of foreign shareholders regarding labor acquisition, market capture, and pollution emissions may further exacerbate the adverse impact of overseas sales by Chinese corporates on their profit margins.

However, foreign shareholders face a greater degree of information asymmetry compared to domestic shareholders due to differences in geography, culture, politics, and other factors [44,45]. This is primarily because they have a poorer understanding of China’s politics, economy, law, and even listed companies. Additionally, the communication cost between foreign shareholders and domestic shareholders as well as management is higher. Consequently, these factors contribute to the outsider disadvantage experienced by foreign shareholders [27,46]. Moreover, driven by profit motives [47] and influenced by the theory of comparative advantage [48], foreign shareholders may resort to transferring profits in order to achieve their profit-seeking goals [49]. Therefore, based on information asymmetry and profit-seeking motives mentioned earlier, overseas sales can be seen as an effective strategy employed by foreign shareholders for risk aversion and financial speculation. However, it should be noted that such actions can exacerbate the negative impact of overseas sales on corporate profit margins.

Compared to alternative approaches, overseas sales serve as a crucial means for foreign shareholders to mitigate risks and engage in financial speculation, offering the following advantages. Firstly, foreign shareholders possess inherent informational superiority concerning overseas markets, consumers, and even sales channels [43]. This advantage enables them to leverage their knowledge in overseas sales activities for risk avoidance and more convenient financial speculation. Simultaneously, domestic shareholders’ understanding of overseas markets significantly lags behind that of their foreign counterparts, exacerbating the information asymmetry between domestic and foreign shareholders regarding overseas sales. Consequently, this asymmetry facilitates risk avoidance and financial speculation by foreign shareholders through engaging in overseas sales. Secondly, foreign shareholders are driven by the pursuit of cash dividends, which have become a significant means to mitigate risks and financial speculation [27,46]. However, in comparison to cash dividends, overseas sales enable direct profit transfer instead of sharing corporate profits with domestic shareholders. This allows foreign shareholders to attain higher profits. According to transfer pricing theory [50], corporations with foreign shareholders can sell their products at lower prices through overseas sales, particularly to affiliated companies abroad, thereby facilitating the direct transfer of profits from corporations with foreign shareholders to overseas entities. Consequently, corporate profits decrease and dividends cannot be distributed adequately, resulting in increased profitability for foreign shareholders [51]. Therefore, based on motivations related to risk avoidance and financial gains, foreign shareholders exacerbate the negative impact of overseas sales on corporate profit margins.

In conclusion, considering the motives of foreign shareholders such as labor acquisition, risk avoidance, and financial speculation, it can be argued that foreign shareholders exacerbate the adverse impact of overseas sales on corporate profit margins. Therefore, this study proposes research Hypothesis 2.

H2: Foreign shareholder exacerbates the negative impact of overseas sales on corporate profit margins.

## 3. Research design

### 3.1 Sample and data source

We utilize the CSMAR database (China Stock Market and Accounting Research Database) developed by Chinese Shenzhen Xishma Data Technology Co., Ltd. to acquire the necessary financial information of diverse companies. This comprehensive database encompasses corporate financial statements, shareholder particulars, overseas direct investment data, and more. Our research sample commences from June 30, 2003, which marks the initiation of our foreign shareholder information data collection. While overseas sale data is available starting from December 31, 2003. Consequently, our sample period spans from 2003 to 2021 and includes companies listed on the main board and small and medium-sized board of Shanghai Stock Exchange as well as Shenzhen Stock Exchange.

However, considering specific accounting standards and regulations applicable to financial industry firms along with disparities between B shares and A shares in terms of trading methods and purchasing objects among other aspects, which may potentially impact the accuracy and reliability of our research conclusions. Henceforth, we have excluded such financial industry companies as well as B-share companies during our research process. Following meticulous screening procedures mentioned above, our study ultimately encompasses a total of 43,933 company-year samples derived from 4,624 individual companies. Additionally, in order to mitigate potential influence stemming from outliers on research outcomes, we conducted tail-narrowing processing at both the lower (1%) and upper (99%) levels for all continuous variables utilized within this study framework.

### 3.2 Definition of variables

#### 3.2.1 Foreign shareholders (*Foreign*)

The CSMAR database serves as a crucial data source in the realm of Chinese financial research. Its shareholder information is both comprehensive and detailed, encompassing not only the names and shareholdings of listed companies’ top ten shareholders but also providing insights into their nature and other pertinent details. This resource greatly facilitates our access to information regarding foreign shareholders.

On this basis, this study will employ two dimensions to assess the influence of foreign shareholders in Chinese listed companies: whether foreign shareholders hold shares and the proportion of their shareholding. According to China’s Company Law, shareholders who possess more than one-tenth of the voting rights are entitled to propose extraordinary shareholders’ meetings. Therefore, in order to ensure the impact of foreign shareholders on decision-making in Chinese enterprises, we introduce a variable called *ForeignDum* (whether foreign shareholders hold shares), which is assigned a value of 1 when the shareholding ratio of foreign shareholders among the top ten exceeds or equals 10%, and 0 otherwise. Conversely, we operationalize the variable of foreign shareholders’ shareholding ratio *(ForeignRatio*) as the aggregate number of shares held by foreign shareholders among the top ten shareholders, divided by the total share capital of the company. By employing these two variables, we can more precisely assess the position and impact of foreign shareholders in Chinese firms.

#### 3.2.2 Overseas sales of corporations (*OverseasSale*)

Drawing upon the financial data extracted from the CSMAR database, specifically from the overseas business income table, we have calculated the ratio of overseas sales (*OverseasSale*) as a proportion of the total sales income. This approach is in line with the methodology proposed by Su and Hong [3], which is aimed at shedding light on the significance of oversea sales in the context of a company’s overall performance. By examining the ratio of overseas sales to total sales, we can obtain a comprehensive understanding of the extent to which a company’s revenue is derived from international markets, and assess its dependency on these markets.

#### 3.2.3 Corporate Profit Margin (*OperatingMargin*)

The present study primarily relies on the income statements accessible in the CSMAR database to extract pertinent information concerning the operating profit and main business income of enterprises. This information is of paramount importance as it provides a deep understanding of the financial health and performance of these enterprises. To counteract the potential interference of other extraneous factors on the profit margin, we have chosen to utilize the operating profit margin as our primary metric. This is done by dividing the operating profit by the main business income of the enterprises (*OperatingMargin*), a method which has been previously employed by Wang et al. [52] in their research. By adopting this approach, we aim to create a standardized and comparable analysis that solely focuses on the operating profit margin, thereby rendering our findings more robust and reliable. Furthermore, this method allows us to isolate the impact of the main business income on the operating profit margin, enabling us to draw more accurate conclusions about the financial performance of these enterprises.

#### 3.2.4 Control variable

We refer to previous research [53,54], wherein the following variables were rigorously controlled: corporate size (*Size*), represented by the natural logarithm of total assets to reflect overall company size; corporate asset-liability ratio (*Leverage*), calculated as total liabilities divided by total assets, used to measure capital structure; corporate fixed assets ratio (*Capital*), obtained by dividing total fixed assets by total assets to indicate the proportion of fixed assets in the company; corporate market -to- book ratio (*TobinQ*), representing the ratio of market value to book value and utilized for assessing market performance; product market competition (*Competition*), determined as the natural logarithm of the number of companies in the industry, reflecting market competitiveness; Age of corporation (*FirmAge*), derived from taking the natural logarithm of current year minus listing year plus 1, indicating company age; Management shareholding (*ManagerShare*), computed as management-held shares divided by total shares outstanding, reflecting management’s ownership stake in the company; Percentage of independent directors (*BoardIndep*), denoting the proportion of independent directors relative to all directors and serving as an indicator for board independence; Whether chairman and general manager positions are held by same person or not (*Dual*), assigned a value of 1 when both roles are held by one individual and 0 otherwise, measuring characteristics related to corporate governance structure. Board size (*BoardSize*) was measured using natural logarithm transformation on total number of board members within a firm, providing insights into board size. Nature of ownership (*Soe*) was coded as 1 if actual control over a firm is state-owned and 0 otherwise, capturing ownership nature. These variables are considered crucial factors in the study, as they possess the potential to impact the firm’s profit margin. By effectively controlling and incorporating these variables into our research model, we can enhance the precision of evaluating the economic implications of foreign shareholders on the firm. The definitions for all variables are provided in Table 1.

**Table 1:** Variable definitions.

Variable	Definition
*OperatingMargin*	Operating profit scaled by main business revenue.
*OverseasSale*	Overseas sales divided by total sales.
*ForeignDum*	When the proportion of shares held by foreign shareholders is greater than or equal to 10%, the value is one, otherwise the value is zero.
*ForeignRatio*	The percentage of common shares owned by foreign shareholder.
*Size*	Natural logarithm of total assets.
*Leverage*	Total debts over book value of total assets.
*Capital*	Net plant, property, and equipment scaled by total assets.
*TobinQ*	The market value of equity over the book value of equity.
*Competition*	The natural logarithm of the total number of corporates in an industry.
*FirmAge*	Natural logarithm of 1 plus the number of years since a firm’s first appearance in the CSMAR stock return file.
*ManagerShare*	The percentage of common shares owned by executives.
*BoardIndep*	The number of independent directors scaled by the total number of directors.
*Dual*	Equal one if the chairman and the general manager positions are held by the same person and zero otherwise.
*BoardSize*	The natural logarithm of the total number of directors.
*Soe*	Equal one for state-owned forms and zero otherwise.

### 3.3 Research design

Building upon the discussion on the impact of China’s overseas sales on corporate profit margins, we conduct a further analysis to examine how the characteristics of foreign shareholders influence the relationship between China’s overseas sales and corporate profit margins. To investigate this, we employ the following regression model:

$$\begin{array}{c} OperatingMargin=\beta_{0}+\beta_{1}OverseasSale+\beta_{x}Controls\\ +\lambda +\gamma +\eta +\mu \end{array}$$ (1)

The dependent variable, *OperatingMargin*, represents the corporate profit margin and is measured as the operating profit rate by dividing operating profit with main business revenue, following Wang et al. [52]. The explanatory variable *OverseasSale* denotes corporate overseas sales and is defined as the ratio of overseas sales to total sales, based on Su and Hong [3]. *Controls* refer to a vector of firm-level control variables including corporate size, asset-liability ratio, fixed assets ratio, market -to- book ratio, etc. Individual fixed effects for industry (λ), time (γ), and province (ƞ) are considered along with random disturbances denoted by μ. The coefficient β_1_ is the influence of overseas sales on corporate profit margins. In the case of a negative value for β_1_, it implies that Chinese enterprises experience diminishing profit margins as their overseas sales increase. Conversely, a positive value of β_1_ indicates that the profit margin of Chinese enterprises exhibits an upward trend in response to increasing overseas sales.

The following regression model is employed to assess the impact of foreign shareholders on the association between overseas sales and profit margins, thereby testing our research hypothesis:

$$\begin{array}{l} OperatingMargin=\alpha_{0}+\alpha_{1}OverseasSale+\alpha_{2}Foreign\\ \;+\alpha_{3}Foreign\times OverseasSale+\alpha_{x}Controls\\ \;+\lambda +\gamma +\eta +\mu \end{array}$$ (2)

The dependent variable, *OperatingMargin*, represents the profit margin of the corporation, while the independent variable, *OverseasSale*, denotes corporate sales overseas. The key explanatory variables Foreign consist of whether foreign shareholders hold shares (*ForeignDum*) and the proportion of shares owned by foreign shareholders (*ForeignRatio*). Additionally, a vector of firm-level control variables including corporate size, asset-liability ratio, fixed assets ratio, market -to- book ratio and others are considered as controls. Furthermore, individual fixed effects for industry (λ), time (γ), and province (*ƞ*) are incorporated along with random disturbances denoted by μ.

The coefficient α3 represents the impact of foreign shareholders on both overseas sales and corporate profit margins. A significantly positive value for α3 suggests that foreign shareholders mitigate the negative effect of overseas sales on corporate profit margins, thereby supporting Hypothesis 1. Conversely, a significantly negative value for α3 indicates that foreign shareholders exacerbate the adverse impact of overseas sales on corporate profit margins, thus supporting Hypothesis 2.

## 4. Empirical results

### 4.1 Descriptive statistical analysis

The descriptive statistics of the main variables are presented in Table 2. In Panel A, the statistical findings reveal that the mean value of corporate profit margin, *OperatingMargin*, is 0.078 yuan per unit sale. This indicates that each unit sale generates a profit of 0.078 yuan. On average, overseas sales (*OverseasSale*) account for 10.1% of the total corporate sales revenue. Furthermore, *ForeignDum* has a mean value of 0.118, suggesting that more than 10% of the sample consists of firms with foreign shareholders. Additionally, foreign shareholders own approximately 3.8% of common shares in these firms. The remaining control variables exhibit similar characteristics to those observed in previous studies.

**Table 2:** The descriptive statistics of the main variables.

Panel A									
	N	Mean	SD	Min	P25	Median		P75	Max
***OperatingMargin***	**43933**	**0.078**	**0.191**	**-0.959**	**0.025**	**0.076**		**0.154**	**0.580**
***OverseasSale***	**43933**	**0.101**	**0.199**	**0.000**	**0.000**	**0.000**		**0.102**	**0.892**
***ForeignDum***	**43933**	**0.118**	**0.323**	**0.000**	**0.000**	**0.000**		**0.000**	**1.000**
***ForeignRatio***	**43933**	**0.038**	**0.103**	**0.000**	**0.000**	**0.000**		**0.006**	**0.596**
*Size*	43933	22.007	1.279	19.689	21.076	21.817		22.722	26.039
*Leverage*	43933	0.431	0.205	0.052	0.268	0.429		0.587	0.884
*Capital*	43933	0.224	0.168	0.002	0.092	0.190		0.321	0.719
*TobinQ*	43933	0.004	0.003	0.001	0.002	0.003		0.004	0.019
*Competition*	43933	5.748	1.241	2.485	4.719	6.004		6.798	7.520
*FirmAge*	43933	1.992	0.911	0.000	1.386	2.197		2.708	3.296
*ManagerShare*	43933	0.117	0.191	0.000	0.000	0.001		0.186	0.681
*BoardIndep*	43933	0.371	0.053	0.273	0.333	0.333		0.429	0.571
*Dual*	43933	0.252	0.434	0.000	0.000	0.000		1.000	1.000
*BoardSize*	43933	2.145	0.205	1.609	1.946	2.197		2.197	2.708
*SOE*	43933	0.399	0.490	0.000	0.000	0.000		1.000	1.000
Panel B									
	Sample with Foreign Shareholder (N = 5188)			Sample without Foreign Shareholder (N = 38745)			Differences		
***OverseasSale***	**0.173**			**0.091**			0.082*** (**27.950**)		
***OperatingMargin***	**0.096**			**0.076**			0.020*** (**7.260**)		

Note: This table reports summary statistics. All dates are sourced from CSMAR database. All variables are defined in the Table 1. The t-statistics are reported in parentheses

*, **, and *** indicate statistical significance at the level of 10%, 5% and 1%, respectively.

The results of the mean test in Panel B of Table 2 indicate that the sample with foreign shareholders exhibits significantly higher levels of overseas sales compared to the sample without foreign shareholders. Furthermore, there is a noticeable disparity in corporate profit margins between these two samples.

### 4.2 Baseline regression results

Table 3 presents the relationship between foreign shareholders, overseas sales, and corporate profit margins. Column (1) examines the impact of overseas sales on corporate profit margin, while columns (2) and (3) investigate whether foreign shareholders holding shares can mitigate the negative impact of overseas sales on corporate profit margins. Columns (4) and (5) explore how the percentage of shares held by foreign shareholders affects overseas sales and corporate profit margins. The regression results in column (1) reveal a significantly negative coefficient for overseas sales at the level of 1%, indicating that Chinese enterprises experience a detrimental effect on their profit margin due to overseas sales. However, columns (2), (3), (4), and (5) show that both *ForeignDum×OverseasSale* and *ForeignRatio×OverseasSale* have significantly positive coefficients at either 1%. These findings suggest that when foreign shareholders hold more shares or own a higher percentage of shares in Chinese enterprises, they can help alleviate the adverse effects caused by overseas sales on corporate profits margins.

**Table 3:** The relationship between foreign shareholder, overseas sales and corporate profit margin.

	*OperatingMargin*				
	(1)	(2)	(3)	(4)	(5)
***ForeignDum×OverseasSale***		0.049***	0.057***		
		**(4.59)**	**(5.43)**		
***ForeignRatio×OverseasSale***				0.123***	0.154***
				**(3.18)**	**(4.00)**
***OverseasSale***	-0.021***	-0.039***	-0.029***	-0.038***	-0.028***
	**(-5.17)**	(-8.38)	(-6.13)	(-8.00)	(-5.77)
*ForeignDum*		-0.018***	-0.024***		
		(-5.49)	(-7.34)		
*ForeignRatio*				-0.029***	-0.046***
				(-3.08)	(-4.84)
*Size*	0.053***	0.048***	0.055***	0.048***	0.054***
	(54.27)	(55.04)	(53.93)	(55.14)	(54.02)
*Leverage*	-0.433***	-0.395***	-0.435***	-0.394***	-0.434***
	(-67.86)	(-66.27)	(-68.18)	(-66.08)	(-67.98)
*Capital*	-0.085***	-0.044***	-0.084***	-0.044***	-0.084***
	(-13.07)	(-7.97)	(-12.91)	(-8.08)	(-12.96)
*TobinQ*	5.992***	3.717***	6.081***	3.694***	6.044***
	(11.98)	(8.53)	(12.16)	(8.47)	(12.08)
*Competition*	-0.013**	-0.015***	-0.013**	-0.015***	-0.013**
	(-2.17)	(-18.84)	(-2.20)	(-18.92)	(-2.19)
*FirmAge*	-0.025***	-0.029***	-0.026***	-0.029***	-0.025***
	(-21.55)	(-25.29)	(-22.06)	(-25.10)	(-21.78)
*ManagerShare*	0.057***	0.034***	0.054***	0.036***	0.056***
	(11.32)	(6.65)	(10.38)	(7.04)	(10.78)
*BoardIndep*	-0.069***	-0.089***	-0.067***	-0.089***	-0.068***
	(-3.64)	(-4.57)	(-3.57)	(-4.59)	(-3.60)
*Dual*	-0.001	-0.003	-0.001	-0.003	-0.001
	(-0.29)	(-1.61)	(-0.31)	(-1.64)	(-0.36)
*BoardSize*	-0.004	0.010*	-0.003	0.010*	-0.003
	(-0.65)	(1.90)	(-0.60)	(1.86)	(-0.64)
*SOE*	0.015***	0.020***	0.014***	0.020***	0.014***
	(6.63)	(9.24)	(6.25)	(9.36)	(6.39)
*Constant*	-0.819***	-0.671***	-0.841***	-0.662***	-0.830***
	(-26.29)	(-30.14)	(-26.91)	(-29.88)	(-26.60)
*Industry*	YES	NO	YES	NO	YES
*Year*	YES	NO	YES	NO	YES
*Province*	YES	YES	YES	YES	YES
*N*	43933	43933	43933	43933	43933
*R* ^*2*^	0.232	0.188	0.233	0.188	0.232
*Adj*. *R*^*2*^	0.230	0.188	0.231	0.188	0.231

Note: This table reports the relationship between foreign shareholder, overseas sales and corporate profit margin. All dates are sourced from CSMAR database. All variables are defined in the Table 1. The t-statistics are reported in parentheses

*, **, and *** indicate statistical significance at the level of 10%, 5% and 1%, respectively.

### 4.3 Analysis of dynamic adjustment of foreign shareholders

Foreign shareholders may prefer to collaborate with companies that have higher profit margins. Therefore, we examine the adjustments in overseas sales and corporate profit margins from a dynamic perspective before foreign shareholders enter, during their tenure, and after their withdrawal. To investigate the influence of foreign shareholders on overseas sales and corporate profit margins comprehensively, we select companies with foreign shareholders as the experimental group and those without any history of foreign shareholder involvement as the control group. We define a variable called *Before_ForeignDum* to represent the period before foreign shareholders enter. It takes a value of one if the sample belongs to the experimental group during this period, otherwise it is zero. Similarly, we define another variable called *Current_ForeignDum* to indicate whether or not a sample belongs to the experimental group during the tenure of foreign shareholders. It takes a value of one if so, otherwise it is zero. Lastly, we define *After_ForeignDum* as a variable representing the period after foreign shareholder withdrawal. Its value is one when samples belong to the experimental group during this phase and zero otherwise.

The dynamic changes of overseas sales and corporate profit margin are reported in Table 4. Columns (1) to (3) present the regression results for three variables: *Before_ForeignDum*, *Current_ForeignDum*, and *After_ForeignDum*, respectively. Column (4) displays the regression result when all three variables are included simultaneously. These findings indicate that only the coefficient of the interaction term between *Current_ForeignDum* and *FSTS* consistently exhibits a significant positive effect at a 1% significance level. This implies that foreign shareholders effectively mitigate the adverse impact of overseas sales on profit margins. However, there is no significant alleviating effect observed before foreign shareholders enter or after foreign shareholder withdrawal regarding the adverse impact of overseas sales on profit margins. These results provide further support for Hypothesis 1.

**Table 4:** Dynamic changes of overseas sales and profit margin.

	*OperatingMargin*			
	(1)	(2)	(3)	(4)
***Before_ForeignDum×OverseasSale***	-0.021***			**-0.008**
	**(-2.72)**			**(-0.84)**
***Current_ForeignDum×OverseasSale***		0.057***		0.049***
		**(5.43)**		**(3.91)**
***After_ForeignDum×OverseasSale***			-0.024*	**-0.016**
			**(-1.75)**	**(-1.19)**
*OverseasSale*	-0.010*	-0.029***	-0.019***	-0.022***
	(-1.78)	(-6.13)	(-4.31)	(-2.93)
*Before_ForeignDum*	0.003			-0.004*
	(1.50)			(-1.75)
*Current_ForeignDum*		-0.024***		-0.026***
		(-7.34)		(-6.91)
*After_ForeignDum*			0.008***	0.005*
			(3.07)	(1.82)
*Size*	0.054***	0.055***	0.054***	0.054***
	(53.16)	(53.93)	(54.26)	(53.35)
*Leverage*	-0.433***	-0.435***	-0.433***	-0.434***
	(-67.48)	(-68.18)	(-67.89)	(-67.77)
*Capital*	-0.085***	-0.084***	-0.085***	-0.084***
	(-13.04)	(-12.91)	(-13.10)	(-12.96)
*TobinQ*	6.000***	6.081***	6.016***	6.047***
	(11.98)	(12.16)	(12.02)	(12.07)
*Competition*	-0.013**	-0.013**	-0.013**	-0.013**
	(-2.23)	(-2.20)	(-2.14)	(-2.12)
*FirmAge*	-0.025***	-0.026***	-0.025***	-0.026***
	(-21.39)	(-22.06)	(-21.50)	(-21.93)
*ManagerShare*	0.058***	0.054***	0.057***	0.055***
	(11.25)	(10.38)	(11.36)	(10.54)
*BoardIndep*	-0.069***	-0.067***	-0.069***	-0.068***
	(-3.62)	(-3.57)	(-3.66)	(-3.58)
*Dual*	-0.001	-0.001	-0.001	-0.001
	(-0.32)	(-0.31)	(-0.32)	(-0.34)
*BoardSize*	-0.003	-0.003	-0.004	-0.003
	(-0.64)	(-0.60)	(-0.67)	(-0.60)
*SOE*	0.015***	0.014***	0.014***	0.014***
	(6.66)	(6.25)	(6.51)	(6.20)
*Constant*	-0.823***	-0.841***	-0.820***	-0.832***
	(-26.25)	(-26.91)	(-26.32)	(-26.55)
*Industry*	YES	NO	YES	YES
*Year*	YES	NO	YES	YES
*Province*	YES	YES	YES	YES
*N*	43933	43933	43933	43933
*R* ^*2*^	0.232	0.233	0.232	0.233
*Adj*. *R*^*2*^	0.230	0.231	0.230	0.231

Note: This table reports dynamic changes of overseas sales and profit margin. All dates are sourced from CSMAR database. *Before_ForeignDum* is the variable before foreign shareholder enter. *Current_ForeignDum* is the variable foreign shareholder stay. *After_ForeignDum* is the variable after foreign shareholder withdrawal. Other variables are defined in the Table 1. The t-statistics are reported in parentheses

*, **, and *** indicate statistical significance at the level of 10%, 5% and 1%, respectively.

### 4.4 Robustness test

#### 4.4.1 Propensity score matching method

Corporate characteristics may have an impact on foreign shareholders. To mitigate this effect, we employed the propensity score matching method to control for corporate characteristics and avoid any potential bias in our research findings. Specifically, we selected a sample of firms with foreign shareholders as the experimental group and matched them with a control group of firms without foreign shareholders using 1:1 nearest neighbor matching based on cash dividend, corporate debt ratio, corporate growth, and long-term investment. Table 5 presents the inter-group differences between variables before and after propensity score matching, which demonstrate that significant differences existed between the two groups prior to matching but were eliminated after matching.

**Table 5:** Disparities in variables pre- and post-propensity score matching.

	Before Matching			After Matching		
	Sample with foreign shareholder	Sample without foreign shareholder	T test	Sample with foreign shareholder	Sample without foreign shareholder	T test
	(1)	(2)	(3)	(4)	(5)	(6)
*CashDividend*	0.173	0.097	0.076*** (31.390)	0.179	0.177	0.002 (0.621)
*Leverage*	0.428	0.443	-0.015*** (4.500)	0.428	0.425	0.003 (0.903)
*Growth*	0.179	0.192	-0.013* (-1.838)	0.175	0.180	-0.005 (-0.576)
*LnInvest*	17.326	16.858	0.468*** (4.581)	17.361	17.268	0.093 (0.671)

Note: This table presents disparities in variables before and after propensity score matching. All dates are sourced from the CSMAR database. *CashDividend* represents the cash dividend per share in the previous year (t-1). *Leverage* indicates the ratio of total debts to book value of total assets. *Growth* measures the percentage change in sales revenue from year t to t-1, relative to sales revenue in t-1. *LnInvest* denotes the natural logarithm of corporate long-term investment. The t-statistics are reported in parentheses

*, **, and *** indicate statistical significance at the level of 10%, 5% and 1%, respectively.

We further estimate the actual impact of foreign shareholders on overseas sales and corporate profit margins, and the results are presented in Table 6. The coefficients of *ForeignDum×OverseasSale* in columns (1) and (2) exhibit significant positive effects at a significance level of 1%. Additionally, the coefficients of *ForeignRatio×OverseasSale* in columns (3) and (4) demonstrate positive and statistically significant effects at least at a significance level of 10%. These findings align with our previous analysis, thus ensuring the robustness of our research conclusions.

**Table 6:** Robustness test of propensity score matching method.

	*OperatingMargin*			
	(1)	(2)	(3)	(4)
***ForeignDum×OverseasSale***	0.040***	0.041***		
	**(2.85)**	**(2.87)**		
***ForeignRatio×OverseasSale***			0.064*	0.071**
			**(1.88)**	**(2.04)**
*OverseasSale*	-0.050***	-0.038***	-0.039***	-0.027***
	(-4.24)	(-3.19)	(-4.00)	(-2.72)
*ForeignDum*	-0.020***	-0.023***		
	(-4.78)	(-5.44)		
*ForeignRatio*			-0.025**	-0.033***
			(-2.41)	(-3.14)
*Size*	0.041***	0.047***	0.040***	0.045***
	(26.71)	(26.13)	(27.05)	(26.48)
*Leverage*	-0.407***	-0.428***	-0.404***	-0.425***
	(-34.23)	(-33.97)	(-34.00)	(-33.77)
*Capital*	-0.077***	-0.129***	-0.079***	-0.131***
	(-6.95)	(-10.41)	(-7.19)	(-10.54)
*TobinQ*	7.504***	9.370***	7.436***	9.250***
	(8.72)	(9.96)	(8.65)	(9.86)
*Competition*	-0.010***	-0.001	-0.010***	0.000
	(-6.15)	(-0.04)	(-6.36)	(0.01)
*FirmAge*	-0.021***	-0.019***	-0.020***	-0.019***
	(-7.81)	(-7.07)	(-7.52)	(-6.82)
*ManagerShare*	0.013	0.026***	0.018*	0.031***
	(1.38)	(2.71)	(1.95)	(3.30)
*BoardIndep*	-0.125***	-0.102***	-0.127***	-0.103***
	(-3.24)	(-2.67)	(-3.28)	(-2.68)
*Dual*	-0.002	0.001	-0.002	0.001
	(-0.59)	(0.26)	(-0.65)	(0.19)
*BoardSize*	-0.006	-0.021*	-0.007	-0.022*
	(-0.52)	(-1.81)	(-0.58)	(-1.87)
*SOE*	0.017***	0.005	0.018***	0.006
	(3.90)	(1.17)	(4.19)	(1.43)
*Constant*	-0.489***	-0.701***	-0.464***	-0.675***
	(-12.23)	(-10.18)	(-11.74)	(-9.79)
*Industry*	NO	YES	NO	YES
*Year*	NO	YES	NO	YES
*Province*	NO	YES	NO	YES
*N*	9375	9375	9375	9375
*R* ^*2*^	0.207	0.264	0.205	0.262
*Adj*. *R*^*2*^	0.206	0.257	0.204	0.255

Note: This table reports robustness test of propensity scores matching method. All dates are sourced from CSMAR database. All variables are defined in the Table 1. The t-statistics are reported in parentheses

*, **, and *** indicate statistical significance at the level of 10%, 5% and 1%, respectively.

#### 4.4.2 Heckman two-stage estimation method

There may exist self-selection bias between foreign shareholders and corporate profit margins, as foreign shareholders are more likely to invest in corporations with higher profit margins. To address this issue, we employ the Heckman two-stage model. In the first stage, a probit model is constructed to examine factors influencing foreign shareholder participation, such as cash dividends and long-term investments by corporations. In the second stage, the obtained *IMR* value from the first stage is incorporated into Model (2) for regression analysis. The results of the Heckman two-stage regression analysis are presented in Table 7. These findings indicate that *IMR* is statistically significant at a 1% level, suggesting the presence of self-selection bias problem. However, even after controlling for this bias problem, the coefficients of interaction terms—*ForeignDum×OverseasSale* and *ForeignRatio×OverseasSale*—remain significantly positive at a 1% level. This implies that foreign shareholders mitigate the negative impact of overseas sales on profit margins, thus confirming robustness of our research conclusions.

**Table 7:** Robustness test of the Heckman two-stage estimation method.

	*ForeignDum*	*OperatingMargin*	
	The first stage	The second stage	
	(1)	(2)	(3)
***ForeignDum×OverseasSale***		0.066***	
		**(5.93)**	
***ForeignRatio×OverseasSale***			0.185***
			**(4.56)**
*OverseasSale*		-0.024***	-0.023***
		(-4.70)	(-4.37)
*ForeignDum*		-0.023***	
		(-6.87)	
*ForeignRatio*			-0.044***
			(-4.55)
*CashDividend*	0.339***		
	(6.78)		
*LnInvest*	-0.007***		
	(-3.96)		
*Size*	0.317***	-0.054***	-0.055***
	(28.89)	(-13.65)	(-13.83)
*Leverage*	-0.624***	-0.179***	-0.178***
	(-10.61)	(-15.33)	(-15.17)
*Capital*	0.558***	-0.268***	-0.269***
	(8.52)	(-29.98)	(-30.03)
*TobinQ*	21.011***	-2.180***	-2.238***
	(5.64)	(-3.52)	(-3.61)
*Competition*	0.076	-0.039***	-0.039***
	(1.32)	(-6.35)	(-6.35)
*FirmAge*	-0.394***	0.113***	0.114***
	(-26.81)	(22.53)	(22.67)
*ManagerShare*	-1.661***	0.604***	0.607***
	(-23.16)	(29.78)	(29.90)
*BoardIndep*	0.612***	-0.274***	-0.276***
	(3.22)	(-13.13)	(-13.16)
*Dual*	0.049**	-0.018***	-0.018***
	(2.22)	(-7.66)	(-7.72)
*BoardSize*	0.238***	-0.085***	-0.086***
	(4.33)	(-13.59)	(-13.62)
*SOE*	-0.330***	0.125***	0.125***
	(-14.17)	(27.78)	(27.85)
***IMR***		-0.414***	-0.414***
		**(-28.08)**	**(-28.10)**
*Constant*	-9.254***	2.656***	2.675***
	(-27.06)	(20.98)	(21.09)
*Industry*	YES	YES	YES
*Year*	YES	YES	YES
*Province*	YES	YES	YES
*N*	40734	40734	40734
*Pseudo*.*R*^*2*^*/R*^*2*^	0.123	0.237	0.236
*Adj*. *R*^*2*^	-	0.235	0.234

Note: This table reports robustness test of the Heckman two-stage estimation method. All dates are sourced from CSMAR database. *CashDividend* is cash dividend per share in t-1 year. *LnInvest* is the natural logarithm of corporate long-term investment. other variables are defined in the Table 1. The z-statistics or t-statistics are reported in parentheses

*, **, and *** indicate statistical significance at the level of 10%, 5% and 1%, respectively.

#### 4.4.3 Replace explanatory variables

Referring to Johanson and Vahnle [55], we define regular overseas sales (*ROverseasSale*) as follows. If the proportion of a corporation’s overseas sales is less than 5% in at least one year out of three consecutive years, *ROverseasSale* is assigned a value of 1. If a corporation’s overseas sales exceed 5% for three consecutive years, *ROverseasSale* is assigned a value of 2. If a corporation’s overseas sales exceed 5% for three consecutive years and it has established an overseas trading company, *ROverseasSale* is assigned a value of 3. If a corporation’s overseas sales exceed 5% for three consecutive years and it has established an overseas manufacturing subsidiary, *ROverseasSale* is assigned a value of 4. If there are no overseas sales, then *ROverseasSales* equals zero. Additionally, we redefine foreign shareholders as *ForeignDum1* and *ForeignDum2*. When foreign shareholders are present in the corporation, *ForeignDum1* equals one, otherwise it equals zero. Similarly, *ForeignDum2* equals one when the percentage of shares owned by foreign shareholders exceeds or equals to15%, otherwise it equals zero.

Table 8 presents the results of robustness tests conducted by replacing explanatory variables. The findings indicate that regardless of whether we substitute overseas sales, foreign shareholders or both, the coefficients of the interaction term are predominantly positive at a significance level of 1%. These outcomes further reinforce the validity and reliability of our conclusions.

**Table 8:** Robustness tests conducted by replacing explanatory variables.

	*OperatingMargin*					
	(1)	(2)	(3)	(4)	(5)	(6)
***ForeignDum×ROverseasSale***	0.015***					
	**(5.52)**					
***ForeignRatio×ROverseasSale***		0.043***				
		**(3.35)**				
***ForeignDum1×OverseasSale***			0.025***			
			**(3.16)**			
***ForeignDum2×OverseasSale***				0.058***		
				**(5.07)**		
***ForeignDum1×ROverseasSale***					0.006***	
					**(2.74)**	
***ForeignDum2×ROverseasSale***						0.016***
						**(5.52)**
*ROverseasSale*	-0.010***	-0.009***			-0.011***	-0.010***
	(-8.04)	(-7.54)			(-6.96)	(-8.06)
*OverseasSale*			-0.031***	-0.028***		
			(-5.55)	(-5.98)		
*ForeignDum*	-0.026***					
	(-7.39)					
*ForeignRatio*		-0.053***				
		(-4.37)				
*ForeignDum1*			-0.001		-0.001	
			(-0.32)		(-0.32)	
*ForeignDum2*				-0.024***		-0.026***
				(-6.69)		(-7.00)
*Size*	0.055***	0.054***	0.053***	0.054***	0.054***	0.055***
	(54.24)	(54.26)	(51.78)	(53.93)	(52.12)	(54.25)
*Leverage*	-0.435***	-0.434***	-0.432***	-0.435***	-0.431***	-0.434***
	(-68.12)	(-67.93)	(-67.17)	(-68.13)	(-67.11)	(-68.09)
*Capital*	-0.084***	-0.085***	-0.085***	-0.084***	-0.086***	-0.084***
	(-13.01)	(-13.04)	(-13.11)	(-12.87)	(-13.24)	(-12.99)
*TobinQ*	6.098***	6.055***	5.949***	6.050***	5.946***	6.060***
	(12.19)	(12.10)	(11.86)	(12.10)	(11.86)	(12.12)
*Competition*	-0.012**	-0.012**	-0.013**	-0.013**	-0.013**	-0.012**
	(-2.09)	(-2.09)	(-2.22)	(-2.20)	(-2.11)	(-2.09)
*FirmAge*	-0.025***	-0.025***	-0.025***	-0.026***	-0.024***	-0.025***
	(-21.76)	(-21.49)	(-21.45)	(-21.94)	(-21.18)	(-21.65)
*ManagerShare*	0.054***	0.056***	0.059***	0.054***	0.059***	0.054***
	(10.44)	(10.79)	(11.49)	(10.50)	(11.52)	(10.58)
*BoardIndep*	-0.069***	-0.069***	-0.069***	-0.068***	-0.069***	-0.069***
	(-3.63)	(-3.63)	(-3.61)	(-3.58)	(-3.63)	(-3.63)
*Dual*	-0.000	-0.001	-0.001	-0.001	-0.001	-0.001
	(-0.24)	(-0.29)	(-0.34)	(-0.34)	(-0.27)	(-0.28)
*BoardSize*	-0.003	-0.003	-0.004	-0.003	-0.003	-0.003
	(-0.56)	(-0.60)	(-0.65)	(-0.60)	(-0.60)	(-0.56)
*SOE*	0.013***	0.013***	0.015***	0.014***	0.014***	0.013***
	(5.98)	(6.12)	(6.72)	(6.31)	(6.44)	(6.03)
*Constant*	-0.852***	-0.841***	-0.813***	-0.837***	-0.823***	-0.848***
	(-27.23)	(-26.92)	(-25.86)	(-26.78)	(-26.17)	(-27.11)
*Industry*	YES	YES	YES	YES	YES	YES
*Year*	YES	YES	YES	YES	YES	YES
*Province*	YES	YES	YES	YES	YES	YES
*N*	43933	43933	43933	43933	43933	43933
*R* ^*2*^	0.234	0.233	0.232	0.233	0.233	0.234
*Adj*. *R*^*2*^	0.232	0.232	0.230	0.231	0.231	0.232

Note: This table reports robustness tests conducted by replacing explanatory variables. All dates are sourced from CSMAR database. *ROverseasSale* is regular overseas sales. *ForeignDum1* equal one if a corporate has foreign shareholder, otherwise it equal zero. *ForeignDum2* equal one when the percentage of shares owned by foreign shareholders is greater than or equal to 15%, otherwise it equal zero. other variables are defined in the Table 1. The t-statistics are reported in parentheses

*, **, and *** indicate statistical significance at the level of 10%, 5% and 1%, respectively.

#### 4.4.4 Sample adjustment

In order to adapt to the evolving needs of corporate development and changes in the capital market, as well as achieve international convergence of accounting standards for business enterprises, China implemented the Accounting Standards for Business Enterprises on January 1, 2007. To ensure that our findings remain unaffected by significant criteria changes, we have excluded samples prior to 2007 from our analysis. Table 9 presents robustness tests using alternative subsamples. The results in columns (1)—(4) demonstrate a statistically significant positive relationship between the coefficients of interaction terms at a significance level of 1%. Moreover, foreign shareholders mitigate the adverse impact of overseas sales on profit margins, thereby supporting Hypothesis 1. These findings further reinforce the reliability and validity of our conclusions.

**Table 9:** Robustness test with alternative subsample.

	*OperatingMargin*			
	(1)	(2)	(3)	(4)
***ForeignDum×OverseasSale***	0.044***	0.052***		
	**(4.12)**	**(4.94)**		
***ForeignRatio×OverseasSale***			0.103***	0.130***
			**(2.72)**	**(3.47)**
*OverseasSale*	-0.037***	-0.029***	-0.036***	-0.028***
	(-7.83)	(-5.99)	(-7.46)	(-5.60)
*ForeignDum*	-0.015***	-0.021***		
	(-4.40)	(-6.13)		
*ForeignRatio*			-0.019**	-0.035***
			(-2.01)	(-3.57)
*Size*	0.048***	0.054***	0.048***	0.054***
	(51.13)	(50.84)	(51.32)	(50.98)
*Leverage*	-0.394***	-0.433***	-0.393***	-0.432***
	(-62.63)	(-64.00)	(-62.47)	(-63.80)
*Capital*	-0.059***	-0.100***	-0.060***	-0.100***
	(-10.01)	(-14.17)	(-10.11)	(-14.22)
*TobinQ*	3.950***	6.000***	3.923***	5.956***
	(8.82)	(11.95)	(8.76)	(11.87)
*Competition*	-0.014***	-0.006	-0.014***	-0.006
	(-16.57)	(-0.88)	(-16.66)	(-0.89)
*FirmAge*	-0.028***	-0.025***	-0.027***	-0.025***
	(-22.50)	(-20.54)	(-22.31)	(-20.27)
*ManagerShare*	0.039***	0.055***	0.041***	0.057***
	(7.48)	(10.49)	(7.85)	(10.91)
*BoardIndep*	-0.088***	-0.075***	-0.089***	-0.075***
	(-4.30)	(-3.72)	(-4.32)	(-3.75)
*Dual*	-0.002	-0.000	-0.002	-0.000
	(-1.02)	(-0.10)	(-1.03)	(-0.12)
*BoardSize*	0.008	-0.007	0.008	-0.007
	(1.29)	(-1.12)	(1.26)	(-1.15)
*SOE*	0.021***	0.016***	0.021***	0.016***
	(9.01)	(6.73)	(9.12)	(6.87)
*Constant*	-0.680***	-0.828***	-0.671***	-0.816***
	(-28.21)	(-24.06)	(-27.97)	(-23.74)
*Industry*	NO	YES	NO	YES
*Year*	NO	YES	NO	YES
*Province*	NO	YES	NO	YES
*N*	39190	39189	39190	39189
*R* ^*2*^	0.184	0.229	0.183	0.228
*Adj*. *R*^*2*^	0.183	0.227	0.183	0.227

Note: This table reports the regression results with China A-share listings from 2007 to 2021 as the sample. All dates are sourced from CSMAR database. All variables are defined in the Table 1. The t-statistics are reported in parentheses

*, **, and *** indicate statistical significance at the level of 10%, 5% and 1%, respectively.

## 5. Mechanism analysis

The aforementioned empirical findings demonstrate that foreign shareholders can mitigate the detrimental impact of overseas sales on corporate profit margins. However, as mentioned in the theoretical analysis section, foreign shareholders possess two key advantages. Firstly, they can leverage their expertise in production technology to enhance corporate productivity and thereby alleviate the adverse effects of overseas sales on profit margins. Secondly, foreign shareholders can capitalize on their informational advantage regarding overseas sales to mitigate the negative impact on corporate profitability. Consequently, we will conduct tests focusing on both information mechanisms and productivity mechanisms.

### 5.1 Productivity mechanism

Compared to domestic shareholders, foreign shareholders possess advanced production technology, thereby potentially enhancing the productivity of Chinese corporations. Consequently, foreign shareholders can alleviate the negative impact of overseas sales on profit margins through improved corporate productivity. In this study, we employ the unit production cost (*UnitCost*) and total factor productivity (*TFProductivity*), calculated using the OP method proposed by Olley and Pakes [56], as measures to assess the potential productivity effects brought about by foreign shareholders.

The productivity mechanism test results are presented in Table 10. Columns (1) and (2) display the test results of corporate total factor productivity as an intermediary variable, while columns (3) and (4) present the test results of production cost as an intermediary variable. Empirical findings in columns (1) and (2) reveal that the coefficients of *ForeignDum* or *ForeignRatio* exhibit significant positive effects at a significance level of 1%. This supports the notion that foreign shareholders enhance corporate productivity to mitigate the adverse impact of overseas sales on profit margins. Similarly, empirical results in columns (3) and (4) demonstrate that the coefficient of *ForeignDum* or *ForeignRatio* is significantly negative, also at least at a significance level of 1%, further confirming that foreign shareholders alleviate the adverse effects of overseas sales on profit margins by improving corporate productivity.

**Table 10:** Mechanism test conducted on productivity and information.

	*TFProductivity*		*UnitCost*		*UnitExpense*	
	(1)	(2)	(3)	(4)	(5)	(6)
*ForeignDum*	0.014***		-0.013***		0.010***	
	(3.04)		(-4.83)		(6.67)	
*ForeignRatio*		0.053***		-0.047***		0.038***
		(3.83)		(-5.83)		(7.01)
*Size*	0.017***	0.017***	-0.042***	-0.042***	0.001	0.001
	(7.72)	(7.69)	(-33.44)	(-33.63)	(1.54)	(1.44)
*Leverage*	-0.257***	-0.256***	0.352***	0.351***	-0.045***	-0.044***
	(-19.95)	(-19.81)	(42.52)	(42.32)	(-12.69)	(-12.40)
*Capital*	-0.049***	-0.049***	0.066***	0.066***	-0.101***	-0.101***
	(-3.23)	(-3.28)	(6.58)	(6.62)	(-23.67)	(-23.75)
*TobinQ*	14.793***	14.764***	-6.952***	-6.936***	1.661***	1.643***
	(15.59)	(15.55)	(-10.46)	(-10.44)	(6.30)	(6.21)
*ManagerShare*	0.047***	0.048***	-0.102***	-0.102***	0.018***	0.019***
	(4.61)	(4.67)	(-15.47)	(-15.56)	(5.28)	(5.42)
*BoardIndep*	-0.089**	-0.089**	0.095***	0.096***	0.033***	0.032***
	(-2.32)	(-2.34)	(3.71)	(3.72)	(2.81)	(2.79)
*Dual*	-0.007	-0.006	-0.008***	-0.008***	0.001	0.001
	(-1.64)	(-1.61)	(-3.14)	(-3.20)	(1.11)	(1.20)
*BoardSize*	0.013	0.012	-0.005	-0.005	0.014***	0.013***
	(1.09)	(1.06)	(-0.65)	(-0.62)	(3.64)	(3.61)
*SOE*	-0.037***	-0.037***	-0.002	-0.002	-0.009***	-0.009***
	(-8.01)	(-7.93)	(-0.69)	(-0.78)	(-6.79)	(-6.63)
*Constant*	-0.590***	-0.588***	1.700***	1.699***	0.050***	0.051***
	(-9.47)	(-9.48)	(49.04)	(49.16)	(2.90)	(2.99)
*Industry*	YES	YES	YES	YES	YES	YES
*Year*	YES	YES	YES	YES	YES	YES
*Province*	YES	YES	YES	YES	YES	YES
*N*	17376	17376	17376	17376	17376	17376
*R* ^*2*^	0.208	0.208	0.216	0.216	0.202	0.203
*Adj*. *R*^*2*^	0.204	0.204	0.213	0.213	0.198	0.199

Note: This table presents the results of mechanism tests on productivity and information, with all dates sourced from the CSMAR database. The regression analysis only includes the sample with overseas sales. *TFProductivity* is calculated using the OP method proposed by Olley and Pakes [56]. *UnitCost* represents production cost per unit, while *UnitExpense* denotes unit sales expense. Other variables are defined in Table 1. The t-statistics are reported within parentheses, where

*, **, and *** indicate statistical significance at the levels of 10%, 5%, and 1% respectively.

### 5.2 Information mechanism

Foreign shareholders possess an information advantage in overseas markets, enabling them to leverage insights on foreign markets, consumers, and sales channels to mitigate uncertainty and reduce expenses associated with international sales, thereby enhancing profit margins. Consequently, foreign shareholders can alleviate the adverse impact of overseas sales on profitability by minimizing corporate sales expenditures. In this study, we employ sales expenses as a proxy for the information advantage brought by foreign shareholders. A higher unit sales expense indicates a lower level of overseas information provided by foreign shareholders. Therefore, we utilize the *UnitExpense* to quantify the extent of information contributed by foreign shareholders. A larger index value corresponds to a diminished contribution of information from foreign stakeholders.

The results of the information mechanism test are presented in Table 10. It is evident from the findings in columns (5) and (6) that both the coefficient of *ForeignDum* and *ForeignRatio* exhibit a significantly positive relationship. This implies that the information mechanism does not serve as a crucial means for foreign shareholders to mitigate the adverse impact of overseas sales on profit margins.

## 6. Heterogeneity analysis

The heterogeneity of foreign shareholders and firms may exert varying effects on the ability of foreign shareholders to mitigate the adverse impact of overseas sales on corporate profits. Therefore, we also conducted a comprehensive analysis of the heterogeneity among foreign shareholders and firms

### 6.1 Heterogeneity analysis among foreign shareholders

#### 6.1. 1 Duration of ownership by foreign shareholders

The behavior of foreign shareholders may be influenced by the duration of their stay. A longer stay can alleviate speculative and short-sighted motives, thereby potentially mitigating the inverse correlation between overseas sales and corporate profit margins. We introduce a variable called "*LongForeign*" to represent the duration of foreign shareholder presence. The value of *LongForeign* is zero before a foreign shareholder enters or after they withdraw from the corporation. In the first year of their entry, *LongForeign* is assigned a value of one, while in subsequent years it increases incrementally (e.g., two for the second year).

Table 11 presents columns (1) and (2), which demonstrate how the duration of foreign shareholder presence influences the inverse correlation between overseas sales and corporate profit margins. Empirical results indicate that the coefficient for the interaction term *LongForeign×OverseasSale* is significantly positive at a level of 1%, suggesting that longer stays by foreign shareholders are associated with greater alleviation of this inverse correlation.

**Table 11:** Heterogeneity analysis among foreign shareholders.

	*OperatingMargin*			
	Duration of ownership by foreign shareholders		multiple foreign shareholders	
	(1)	(2)	(3)	(4)
***LongForeign×OverseasSale***	0.004***	0.005***		
	**(3.27)**	**(4.58)**		
***MultipleForeign×OverseasSale***			0.021*	0.037***
			**(1.68)**	**(3.11)**
*OverseasSale*	-0.037***	-0.028***	-0.035***	-0.026***
	(-8.00)	(-5.88)	(-7.94)	(-5.68)
*LongForeign*	-0.001***	-0.002***		
	(-4.59)	(-7.36)		
*MultipleForeign*			-0.000	-0.006
			(-0.06)	(-1.64)
*Size*	0.048***	0.055***	0.047***	0.054***
	(54.60)	(53.38)	(54.56)	(53.16)
*Leverage*	-0.394***	-0.436***	-0.392***	-0.433***
	(-66.17)	(-68.12)	(-65.85)	(-67.70)
*Capital*	-0.043***	-0.083***	-0.045***	-0.085***
	(-7.89)	(-12.83)	(-8.16)	(-13.07)
*TobinQ*	3.699***	6.071***	3.663***	5.990***
	(8.49)	(12.15)	(8.38)	(11.94)
*Competition*	-0.015***	-0.013**	-0.015***	-0.013**
	(-18.95)	(-2.20)	(-19.10)	(-2.21)
*FirmAge*	-0.029***	-0.025***	-0.029***	-0.025***
	(-25.14)	(-21.75)	(-25.02)	(-21.49)
*ManagerShare*	0.035***	0.054***	0.037***	0.058***
	(6.76)	(10.56)	(7.33)	(11.31)
*BoardIndep*	-0.087***	-0.065***	-0.090***	-0.069***
	(-4.51)	(-3.45)	(-4.63)	(-3.63)
*Dual*	-0.003	-0.001	-0.003	-0.001
	(-1.62)	(-0.30)	(-1.62)	(-0.30)
*BoardSize*	0.011**	-0.003	0.010*	-0.003
	(1.99)	(-0.51)	(1.85)	(-0.64)
*SOE*	0.020***	0.014***	0.020***	0.015***
	(9.37)	(6.29)	(9.51)	(6.64)
*Constant*	-0.671***	-0.850***	-0.653***	-0.820***
	(-29.93)	(-27.03)	(-29.34)	(-26.12)
*Industry*	YES	YES	YES	YES
*Year*	YES	YES	YES	YES
*Province*	YES	YES	YES	YES
*N*	43933	43933	43933	43933
*R* ^*2*^	0.188	0.226	0.188	0.232
*Adj*. *R*^*2*^	0.188	0.225	0.188	0.230

Note: This table reports heterogeneity analysis among foreign shareholders. All dates are sourced from CSMAR database. *LongForeign* represents the duration of holding by foreign investors, while *MultipleForeign* takes a value of one if the number of foreign shareholders exceeds one, otherwise it is zero. The remaining variables are defined in Table 1. The t-statistics are presented within parentheses, with

*, **, and *** denoting statistical significance at the 10%, 5%, and 1% levels respectively.

#### 6.1.2 Multiple foreign shareholders

Similar to the ownership structure of multiple major shareholders, when there are multiple foreign shareholders, each foreign shareholder will engage in corporate governance based on their individual interests. This can foster mutual supervision and balance among them, thereby reducing the potential for private interests to negatively impact other foreign shareholders. The presence of multiple foreign shareholders creates a system of checks and balances that can restrain behaviors detrimental to corporate profit margins, consequently mitigating the adverse effects of overseas sales on profitability. To capture this concept, we introduce a binary variable called "*MultipleForeign*", which takes a value of one if there is more than one foreign shareholder and zero otherwise.

In Table 11, columns (3) and (4) present the influence of multiple foreign shareholders on mitigating the negative impact of overseas sales on corporate profits. Our empirical findings reveal that the coefficients associated with interaction terms between multiple foreign shareholders and overseas sales are significantly positive at least at a 10% level. This suggests that having multiple foreign shareholders can also alleviate the adverse effects of overseas sales on corporate profit margin.

### 6.2 Heterogeneity analysis among firms

#### 6.2.1 Nature of property rights

From the perspective of property rights, enterprises with different property rights attributes are subject to varying degrees of government intervention and social security responsibilities [57,58]. The presence of these characteristics may influence the role and impact of foreign shareholders, leading to diverse effects on the negative relationship between overseas sales and corporate profit margins. Based on the nature of controlling shareholders, we categorize enterprises into state-owned enterprises (*Soe*) and non-state-owned enterprises (*Non-Soe*), further dividing non-state-owned enterprises into private enterprises *(Private*) and foreign-funded enterprises (*FFE*). Subsequently, Model (2) is applied to each sample for regression analysis.

Table 12 presents heterogeneity analysis based on property right. Columns (1) and (2) illustrate the effect of foreign shareholders on the negative relationship between overseas sales and corporate profit margins in state-owned (*Soe*) and non-state-owned enterprises (*Non-Soe*) respectively. The results indicate that the coefficients for the interaction term between foreign shareholding ratio and overseas sales are -0.087 and 0.105 respectively, with only the latter being significant at a 5% level. This suggests that foreign shareholders primarily mitigate the adverse impact of overseas sales on corporate profits in non-state-owned enterprises. Furthermore, columns (3) and (4) demonstrate how foreign shareholders affect this negative relationship in private enterprises (*Private*) and foreign-funded enterprises (*FFE*) respectively. The results reveal that the coefficients for the interaction term between foreign shareholding ratio and overseas sales are 0.184 and 0.083 respectively, with only former being significant at a 1% level. This indicates that foreign shareholders play a more prominent role in alleviating the detrimental impact of overseas sales on corporate profits in private enterprises.

**Table 12:** Analysis of corporate heterogeneity through the lens of property rights.

	*OperatingMargin*			
	*Soe*	*Non-Soe*	*Private*	*FFE*
	(1)	(2)	(3)	(4)
***ForeignRatio×OverseasSale***	**-0.087**	0.105**	0.184***	**0.083**
	**(-0.98)**	**(2.47)**	**(3.13)**	**(0.93)**
*OverseasSale*	-0.033***	-0.030***	-0.031***	-0.029
	(-3.87)	(-5.00)	(-4.93)	(-1.13)
*ForeignRatio*	-0.100***	-0.007	-0.053**	0.031
	(-6.06)	(-0.57)	(-2.41)	(1.34)
*Size*	0.045***	0.064***	0.066***	0.049***
	(32.13)	(39.60)	(37.59)	(9.95)
*Leverage*	-0.398***	-0.450***	-0.451***	-0.437***
	(-40.68)	(-48.40)	(-45.68)	(-13.76)
*Capital*	-0.110***	-0.088***	-0.097***	-0.036
	(-12.06)	(-8.26)	(-8.46)	(-1.03)
*TobinQ*	4.832***	6.656***	6.564***	7.942***
	(5.30)	(11.12)	(10.29)	(3.97)
*Competition*	-0.009	0.001	0.009	0.038
	(-0.95)	(0.07)	(0.86)	(1.00)
*FirmAge*	-0.015***	-0.030***	-0.032***	-0.013**
	(-7.04)	(-19.28)	(-19.80)	(-2.36)
*ManagerShare*	0.036	0.056***	0.056***	0.046***
	(0.57)	(10.15)	(9.48)	(2.61)
*BoardIndep*	-0.124***	0.003	-0.009	-0.108
	(-4.90)	(0.11)	(-0.30)	(-0.78)
*Dual*	0.006	-0.002	-0.001	-0.002
	(1.37)	(-0.78)	(-0.58)	(-0.25)
*BoardSize*	-0.014*	0.008	0.003	0.040
	(-1.80)	(0.93)	(0.32)	(0.98)
*Constant*	-0.614***	-1.079***	-1.133***	-0.915***
	(-13.89)	(-20.10)	(-19.91)	(-4.22)
*Industry*	YES	YES	YES	YES
*Year*	YES	YES	YES	YES
*Province*	YES	YES	YES	YES
*N*	14187	24995	23006	1989
*R* ^*2*^	0.289	0.218	0.223	0.266
*Adj*. *R*^*2*^	0.285	0.216	0.220	0.237

Note: This table reports heterogeneity analysis of firm through the lens of property rights. All dates are sourced from CSMAR database. All variables are defined in the Table 1. According to the nature of the shareholders, we categorize the samples into state-owned enterprises (*SOE*) and non-state-owned enterprises (*Non-SOE*), further dividing the latter into private enterprises (*Private*) and foreign-funded enterprises (*FFE*). Subsequently, we conduct separate regressions using Model (2). Simultaneously, we include a variable indicating whether foreign capital holds shares (*ForeignDum*) in the regression analysis for state-owned enterprises, non-state-owned enterprises, and private enterprises. Consistently robust conclusions are drawn from these analyses. The t-statistics are reported in parentheses

*, **, and *** indicate statistical significance at the level of 10%, 5% and 1%, respectively.

#### 6.2.2 Institutional quality in firm locations

Due to variations in resources, geographical location, and national policies, the marketization process exhibits significant regional disparities in terms of the government-market relationship, non-state-owned economy development, product market growth, factor market expansion, market intermediary organization advancement, and legal system environment. In comparison to highly marketized regions, enterprises operating in less marketized areas may experience a more pronounced reliance on overseas sales with lower profit margins. Enhancements in the legal system environment are particularly conducive to attracting foreign investment and facilitating its impact. Consequently, foreign shareholders may exert diverse influences on the inverse association between overseas sales and profit margin. According to the research of An et al. [59], we categorize enterprises as low or high marketization degree based on whether their location’s level of marketization (*Market index*) surpasses or equals the annual average degree of marketization respectively. Similarly, we categorize enterprises based on the non-state-owned economic development level (*NS index*) of the region where they are located, distinguishing between those situated in regions with low levels and high levels of non-state-owned economic development, as determined by whether the *NS index* exceeds or equals the annual average level. Furthermore, we divide enterprises into low or high legal system environment categories depending on whether their location’s legal system environment (*MIOLaw index*) surpasses or equals the annual average level. Subsequently, Model (2) was applied to each sample for regression analysis

Table 13 presents the heterogeneity analysis of the institutional environment. Columns (1) and (2) display the regression results for enterprises with low and high levels of marketization, respectively. The findings indicate that foreign shareholders can effectively alleviate the adverse impact of overseas sales on corporate profit margins in both low and high marketization enterprises; however, this mitigating effect is more pronounced in low marketization enterprises. Columns (3) and (4) present the regression results for enterprises with low and high degrees of non-state-owned economic development, respectively. The results demonstrate that foreign shareholders can significantly mitigate the negative influence of overseas sales on corporate profit margins in both types of enterprises, but this effect is more prominent in those with a lower degree of non-state-owned economic development. Columns (5) and (6) exhibit the regression outcomes for enterprises operating under a weak legal system environment versus those operating under a strong legal system environment, respectively. The findings reveal that only in enterprises with better legal mitigation does the coefficient of interaction between foreign shareholders and overseas sales show significant positive effects, indicating that foreign shareholders’ ability to alleviate the negative impact of overseas sales on corporate profit margins is particularly noteworthy when companies operate within an improved legal framework.

**Table 13:** Examining corporate heterogeneity by assessing institutional quality in firm locations.

	*OperatingMargin*					
	*Maket index*		*NS index*		*MIOLaw index*	
	*Low*	*High*	*Low*	*High*	*Low*	*High*
	(1)	(2)	(3)	(4)	(5)	(6)
***ForeignRatio×OverseasSale***	0.209**	0.118***	0.193**	0.113***	**0.099**	0.157***
	**(2.54)**	**(2.91)**	**(2.54)**	**(2.62)**	**(1.59)**	**(3.50)**
*OverseasSale*	-0.038***	-0.023***	-0.060***	-0.015***	-0.015	-0.033***
	(-3.17)	(-4.76)	(-5.06)	(-3.06)	(-1.57)	(-5.98)
*ForeignRatio*	0.007	-0.046***	-0.021	-0.044***	0.012	-0.052***
	(0.35)	(-4.18)	(-1.33)	(-3.54)	(0.83)	(-4.19)
*Size*	0.058***	0.050***	0.054***	0.054***	0.059***	0.049***
	(33.51)	(37.67)	(33.03)	(39.07)	(34.78)	(36.52)
*Leverage*	-0.445***	-0.425***	-0.448***	-0.419***	-0.429***	-0.437***
	(-40.12)	(-51.17)	(-39.97)	(-50.10)	(-40.62)	(-50.51)
*Capital*	-0.087***	-0.114***	-0.095***	-0.111***	-0.092***	-0.113***
	(-8.25)	(-12.07)	(-8.83)	(-11.83)	(-9.27)	(-11.42)
*TobinQ*	4.671***	7.414***	4.754***	7.180***	4.970***	7.408***
	(5.92)	(11.93)	(5.77)	(11.70)	(6.49)	(11.47)
*Competition*	0.004	-0.016**	-0.011	-0.005	0.012	-0.018**
	(0.33)	(-2.07)	(-1.06)	(-0.55)	(1.01)	(-2.24)
*FirmAge*	-0.028***	-0.021***	-0.023***	-0.025***	-0.027***	-0.022***
	(-13.41)	(-14.32)	(-11.43)	(-16.97)	(-13.73)	(-14.08)
*ManagerShare*	0.078***	0.044***	0.081***	0.045***	0.071***	0.045***
	(7.79)	(7.29)	(8.13)	(7.47)	(7.87)	(6.99)
*BoardIndep*	-0.130***	-0.021	-0.161***	0.010	-0.093***	-0.051*
	(-4.26)	(-0.79)	(-5.22)	(0.39)	(-3.14)	(-1.90)
*Dual*	-0.004	0.001	-0.006	0.002	0.002	-0.003
	(-1.08)	(0.53)	(-1.46)	(0.88)	(0.43)	(-0.94)
*BoardSize*	-0.010	-0.002	-0.027***	0.012	-0.007	-0.007
	(-1.18)	(-0.24)	(-3.03)	(1.47)	(-0.81)	(-0.86)
SOE	0.014***	0.017***	0.015***	0.020***	0.016***	0.014***
	(3.85)	(5.47)	(3.92)	(6.48)	(4.67)	(4.37)
*Constant*	-0.895***	-0.706***	-0.720***	-0.937***	-0.971***	-0.632***
	(-15.81)	(-16.08)	(-13.75)	(-19.52)	(-17.22)	(-13.99)
*Industry*	YES	YES	YES	YES	YES	YES
*Year*	YES	YES	YES	YES	YES	YES
*Province*	YES	YES	YES	YES	YES	YES
*N*	16824	22358	16572	22610	17580	21602
*R* ^*2*^	0.232	0.235	0.233	0.236	0.235	0.234
*Adj*. *R*^*2*^	0.228	0.233	0.230	0.233	0.232	0.232

Note: This table presents the results of a heterogeneity analysis of firms based on the institutional quality of their locations. All dates are sourced from the CSMAR database. The variables used in this analysis are defined in Table 1. We categorize firm locations into low and high institutional quality areas using *Market Index*, *NS index*, and *MIOLaw index* as criteria. Subsequently, separate regressions are conducted using Model (2). Additionally, we include a variable indicating whether foreign capital holds shares (*ForeignDum*) in all subsample regression analyses. Consistently robust conclusions are derived from these analyses. The t-statistics are reported in parentheses to indicate statistical significance levels (

* for 10%

** for 5%, and

*** for 1%).

#### 6.2.3 Overseas related party transactions and subsidiaries

In collaboration with foreign shareholders, overseas related party transactions can serve as an effective transfer pricing mechanism [52]. However, such transactions may lead to unfairness in the pricing of overseas sales, resulting in a ’buy high and sell low’ scenario. This unfairness in overseas sales prices is likely to exacerbate the negative impact on corporate profit margins. Therefore, the contribution of foreign shareholders in mitigating the adverse effects of overseas sales on corporate profit margins may primarily manifest in samples without any oversea related party transactions. Meanwhile, overseas subsidiaries can mitigate the adverse impact of overseas sales on corporate profit margins by reducing costs. However, excessive subsidiary presence in corporations with foreign shareholders may result in the formation of multinational conglomerates. Nevertheless, research indicates that multinational conglomerates are more prone to profit shifting through overseas related party transactions [53], thereby diminishing overall profits [60]. Within these multinational conglomerates with foreign shareholders, the latter can directly sell their products to affiliated corporates abroad at lower prices, facilitating the transfer of domestic profits to foreign countries and enabling them to accrue greater private gains. Consequently, foreign shareholders exacerbate the negative effects of overseas sales on corporate profit margins. Therefore, the ability of foreign shareholders to alleviate such adverse impacts is primarily observed in corporations with a limited number of overseas subsidiaries. We categorize the samples into two groups: one without any overseas related party transactions, and another with such transactions. Furthermore, we further divide the samples based on the average number of overseas subsidiaries within their respective industries and years, creating subgroups with a high concentration of overseas subsidiaries and those with a low concentration.

The analysis of corporate heterogeneity through overseas related party transactions and subsidiaries is presented in Table 14. Regression results for columns (1) and (2) are reported, representing the sample without oversea related party transaction and with oversea related party transaction, respectively. The findings reveal a positive coefficient for the interaction term; however, only the former is statistically significant at the 1% level. This suggests that the phenomenon of foreign shareholders mitigating the negative impact of overseas sales on corporate profit margin is primarily observed in samples without overseas related party transactions. Columns (3) and (4) present regression results for samples with many overseas subsidiaries and samples with few overseas subsidiaries, respectively. The results indicate positive coefficients for both interaction terms; nevertheless, only the former exhibits statistical significance at the 1% level. These outcomes suggest that foreign shareholders can alleviate the adverse effects of overseas sales on corporate profit margin mainly in samples characterized by few overseas subsidiaries.

**Table 14:** Analysis of corporate heterogeneity through overseas related party transactions and subsidiaries.

	*OperatingMargin*			
	Overseas related party transactions		Overseas subsidiaries	
	No	Yes	Limited number	Substantial number
	(1)	(2)	(3)	(4)
***ForeignRatio×OverseasSale***	0.141***	**0.145**	0.187***	**0.074**
	**(3.43)**	**(1.58)**	**(2.64)**	**(1.62)**
*OverseasSale*	-0.031***	-0.003	-0.034***	-0.012*
	(-5.75)	(-0.19)	(-3.50)	(-1.89)
*ForeignRatio*	-0.031***	-0.045	-0.021	-0.017
	(-3.06)	(-1.35)	(-1.46)	(-1.33)
*Size*	0.055***	0.040***	0.057***	0.055***
	(48.84)	(12.29)	(35.91)	(35.12)
*Leverage*	-0.432***	-0.409***	-0.415***	-0.449***
	(-61.96)	(-14.84)	(-46.74)	(-42.35)
*Capital*	-0.102***	-0.084***	-0.126***	-0.083***
	(-14.01)	(-3.09)	(-13.78)	(-7.44)
*TobinQ*	5.936***	7.663***	5.761***	6.467***
	(11.49)	(4.32)	(8.81)	(8.22)
*Competition*	-0.006	-0.013	0.007	-0.024**
	(-0.78)	(-0.52)	(0.78)	(-2.23)
*FirmAge*	-0.026***	-0.005	-0.026***	-0.023***
	(-20.54)	(-1.05)	(-16.52)	(-11.73)
*ManagerShare*	0.058***	0.035*	0.052***	0.062***
	(10.68)	(1.66)	(7.10)	(8.13)
*BoardIndep*	-0.066***	-0.036	-0.049*	-0.091***
	(-3.17)	(-0.54)	(-1.85)	(-3.01)
*Dual*	-0.001	0.012	-0.004	0.004
	(-0.56)	(1.56)	(-1.46)	(1.22)
*BoardSize*	-0.006	-0.006	-0.004	-0.012
	(-0.95)	(-0.30)	(-0.55)	(-1.33)
SOE	0.017***	0.002	0.011***	0.015***
	(6.90)	(0.25)	(3.59)	(4.30)
*Constant*	-0.848***	-0.553***	-0.938***	-0.800***
	(-23.51)	(-4.76)	(-19.99)	(-14.82)
*Industry*	YES	YES	YES	YES
*Year*	YES	YES	YES	YES
*Province*	YES	YES	YES	YES
*N*	36759	2423	21763	17419
*R* ^*2*^	0.232	0.247	0.245	0.229
*Adj*. *R*^*2*^	0.230	0.223	0.242	0.226

Note: This table presents the results of a heterogeneity analysis of firms based on the overseas related party transactions and subsidiaries. All dates are sourced from the CSMAR database. The variables used in this analysis are defined in Table 1. According to whether the company engages in overseas transactions, we categorize it into enterprises with international operations and enterprises without international operations. Based on whether the number of overseas subsidiaries exceeds the industry average, we classify them as enterprises with a limited number of overseas subsidiaries or enterprises with a substantial number of overseas subsidiaries. Subsequently, separate regressions are conducted using Model (2). Additionally, we include a variable indicating whether foreign capital holds shares (*ForeignDum*) in all subsample regression analyses. Consistently robust conclusions are derived from these analyses. The t-statistics are reported in parentheses to indicate statistical significance levels (

* for 10%

** for 5%, and

*** for 1%).

#### 6.2.4 Industry attributes

Based on the "pollution paradise" hypothesis, it is believed that environmentally sensitive corporations will relocate from home countries with high environmental protection to developing countries with low environmental protection in order to internalize external costs and seek higher profits. Consequently, China, as a developing country with a low degree of environmental protection, becomes an attractive destination for foreign shareholders due to pollution transfer motivations [61]. This suggests that the motivation for pollution transfer may impact the mitigating effect of foreign shareholders on the negative influence of overseas sales on corporate profit margins. Moreover, research indicates that Chinese enterprises experience reduced profit margins from overseas sales due to factors such as processing trade, factor density, market entry cost, and trade cost [1,2]. Therefore, this phenomenon should be more pronounced in industries characterized by low capital density. As a result, foreign shareholders in enterprises with different levels of capital intensity may exhibit varying effects on the negative relationship between overseas sales and corporate profits. Following industry categorization based on Li and Xiao [62], our samples are divided into non-heavy polluting industries sample and heavy polluting industries sample. Furthermore, enterprises are classified into low capital intensity enterprises or high capital intensity enterprises based on whether their capital intensity exceeds industry average levels. Subsequently, Model (2) is applied to these samples for regression analysis.

The heterogeneity analysis of corporations based on industry attributes is presented in Table 15. Regression results for non-heavily polluting industry enterprises and heavily polluting industry enterprises are reported in columns (1) and (2), respectively. The findings reveal that the coefficients of interaction terms are positive, with only the former being statistically significant at the 5% level. These results suggest that foreign shareholders primarily alleviate the negative effect of overseas sales on corporate profit margins in non-heavy polluting industries sample. Columns (3) and (4) present regression results for low capital intensity enterprises and high capital intensity enterprises, respectively. The results indicate a positive ***coefficient*** of interaction term, but only the former is statistically significant at the 1% level. This demonstrates that foreign shareholders mainly mitigate the negative effect of overseas sales on corporate profit margins in enterprises with low capital intensity.

**Table 15:** Heterogeneity analysis of corporations based on industry attributes.

	*OperatingMargin*			
	Degree of corporate pollution		Capital intensity of enterprises	
	Low	High	Low	High
	(1)	(2)	(3)	(4)
***ForeignRatio×OverseasSale***	0.135**	**0.145**	0.147***	**0.066**
	**(2.31)**	**(0.79)**	**(3.92)**	**(0.59)**
*OverseasSale*	-0.045***	0.011	-0.023***	-0.053***
	(-4.27)	(0.69)	(-4.45)	(-3.84)
*ForeignRatio*	-0.015	-0.065	-0.037***	-0.036
	(-0.83)	(-1.41)	(-3.59)	(-1.56)
*Size*	0.064***	0.060***	0.053***	0.057***
	(26.28)	(15.80)	(43.99)	(23.26)
*Leverage*	-0.472***	-0.451***	-0.422***	-0.465***
	(-33.61)	(-20.56)	(-56.28)	(-30.72)
*Capital*	-0.145***	-0.097***	-0.112***	-0.034**
	(-9.42)	(-4.90)	(-13.23)	(-2.14)
*TobinQ*	4.967***	6.856***	6.421***	3.634***
	(6.80)	(6.94)	(12.71)	(2.60)
*Competition*	-0.000	-0.025	-0.012	0.012
	(-0.04)	(-0.74)	(-1.57)	(0.76)
*FirmAge*	-0.029***	-0.014***	-0.022***	-0.034***
	(-13.94)	(-3.98)	(-16.63)	(-11.70)
*ManagerShare*	0.089***	0.075***	0.052***	0.076***
	(8.20)	(4.07)	(9.27)	(5.44)
*BoardIndep*	-0.035	-0.068	-0.044**	-0.126***
	(-0.97)	(-1.35)	(-2.09)	(-2.74)
*Dual*	0.002	-0.005	-0.000	-0.001
	(0.62)	(-0.87)	(-0.01)	(-0.23)
*BoardSize*	-0.000	-0.021	-0.005	-0.010
	(-0.01)	(-1.19)	(-0.76)	(-0.71)
SOE	0.012**	-0.012	0.014***	0.018***
	(2.55)	(-1.51)	(5.68)	(3.32)
*Constant*	-1.047***	-0.741***	-0.796***	-0.912***
	(-15.11)	(-3.04)	(-20.52)	(-11.70)
*Industry*	YES	YES	YES	YES
*Year*	YES	YES	YES	YES
*Province*	YES	YES	YES	YES
*N*	28181	11001	29478	9704
*R* ^*2*^	0.221	0.258	0.239	0.220
*Adj*. *R*^*2*^	0.159	0.173	0.237	0.214

Note: This table presents the results of a heterogeneity analysis of firms based on the overseas related party transactions and subsidiaries. All dates are sourced from the CSMAR database. The variables used in this analysis are defined in Table 1. We categorize enterprises into two groups: non-polluting enterprises and polluting enterprises, based on their affiliation with polluting industries. Additionally, we classify enterprises as either low capital intensity or high capital intensity, depending on whether their capital intensity exceeds the industry average. Subsequently, separate regressions are conducted using Model (2). Additionally, we include a variable indicating whether foreign capital holds shares (*ForeignDum*) in all subsample regression analyses. Consistently robust conclusions are derived from these analyses. Robust standard errors are clustered at the firm level and t-statistics are reported in parentheses to indicate statistical significance levels (

* for 10%

** for 5%, and

*** for 1%).

## 7. Research conclusion

As a representative developing country, China has emerged as the world’s largest trading nation. However, numerous studies have indicated that in its pursuit of increasing export volume, to some extent, it comes at the expense of corporate profits, leading to the phenomenon of "big but not strong". In light of this observation regarding Chinese enterprises’ reduced profit margins due to overseas sales, we aim to investigate the economic consequences of shareholder characteristics on firms from the perspective of foreign shareholders.

Specifically focusing on Chinese A-share listed companies from 2003 to 2021 as our sample dataset, we investigate the influence of foreign shareholders on the negative relationship between overseas sales and corporate profit margins. Our findings demonstrate that foreign shareholders mitigate the adverse impact of overseas sales on corporate profit margins. This conclusion is supported by a series of robustness tests, including dynamic adjustment analysis for foreign shareholders, propensity score matching regression analysis, Heckman two-stage regression analysis, substitution analyses for explanatory variables, as well as changes in samples. Mechanism analysis reveals that foreign shareholders alleviate the negative impact by enhancing corporate productivity through technology spillover effects. The heterogeneity analysis of foreign shareholders reveals that long-term shareholding and multiple foreign shareholders mitigate the adverse impact of overseas sales on corporate profit margins. Foreign shareholders play a significant role in alleviating the negative effect of overseas sales on corporate profit margins, particularly in non-state-owned enterprises and private enterprises. The phenomenon of foreign shareholders mitigating the negative impact of overseas sales on corporate profit margins is more pronounced in enterprises with low levels of marketization, less non-state-owned economic development, and favorable legal environments. Enterprises without overseas affiliated transactions and those with fewer overseas subsidiaries experience a more substantial reduction in the detrimental effect of overseas sales on their profit margins due to the presence of foreign shareholders. In industries characterized by low pollution levels and lower capital intensity, foreign shareholders have a more significant influence in alleviating the negative impact of overseas sales on corporate profit margins.

The conclusions of this paper may have significant policy implications. Firstly, our findings indicate a negative relationship between overseas sales and enterprise profit margins, which is particularly pronounced in China. Despite China’s active promotion of trade development as a representative of developing countries, this phenomenon suggests that Chinese enterprises may not fully maximize profits during the process of expansion and growth. This conclusion provides valuable empirical evidence for China and other developing countries on how to foster trade while also emphasizing the importance of maintaining healthy profit margins to avoid the "big but not strong" dilemma. Secondly, we observe that foreign shareholders mitigate the adverse impact of overseas sales on corporate profit margins. The volume and profitability derived from international trade are crucial factors determining trade robustness, thus our findings offer empirical evidence for China’s transformation from a trading power to an even stronger one among developing nations. Thirdly, we find that foreign shareholders primarily enhance enterprise productivity through technology spillover effects, thereby alleviating the negative influence of overseas sales on corporate profits. Consequently, when introducing foreign shareholders into domestic enterprises, their technological capabilities play a pivotal role in achieving positive outcomes. This conclusion provides empirical evidence and guidance for other developing countries seeking to attract foreign investors. Furthermore, this study also reveals that prolonged tenure of foreign shareholders and the presence of multiple foreign shareholders can effectively mitigate the adverse impact of overseas sales on firms’ profit margins. Hence, in the process of attracting foreign investment, it is advisable to encourage long-term commitment from foreign shareholders in order to counteract short-sighted effects. Simultaneously, a well-structured composition of foreign shareholdings should be strategically arranged to enhance the positive influence of foreign capital infusion. Lastly, for industries with environmental pollution concerns and those heavily reliant on labor-intensive operations, regulatory measures should be implemented when introducing foreign investment; moreover, enterprises engaged in overseas transactions or possessing numerous overseas subsidiaries must remain vigilant against potential financial speculation by foreign shareholders to safeguard Chinese enterprises’ interests.

## Supporting information

S1 File Code. The empirical test code utilizing Stata16 in the article. Data2003-2021. Requisite data for empirical validation of the article. Psmdata2003-2021. The data obtained after conducting propensity score matching in the article.
(ZIP)
