# Supplementary material for: Foreign shareholder, overseas sale and corporate profit margin
Source: PLoS One. 2024 Feb 5;19(2):e0296021. doi: 10.1371/journal.pone.0296021 (PMC10843053; doi:10.1371/journal.pone.0296021)
Supplement: S1 File — The empirical test code utilizing Stata16 in the article. Data2003-2021. Requisite data for empirical validation of the article. Psmdata2003-2021. The data obtained after conducting propensity score matching in the article. (ZIP) [file pone.0296021.s001.zip › Supporting Information/Code.docx]

*item: Foreign shareholder, overseas sale and corporate profit margin

*new time：2023-10-26

*********************

*table 2-table 4

use data2003-2021,clear

egen miss=rmiss(stkcd fyr opm fsts fs10 fsrate size lev ppe dual indp mshare board lnfirm age tobin soe st ind)

tab miss

drop if miss!=0

drop miss

tab fyr

*winsor

winsor opm,gen(wopm) p(0.01)

winsor fsts,gen(wfsts) p(0.01)

winsor fsrate,gen(wfsrate) p(0.01)

winsor size,gen(wsize) p(0.01)

winsor lev,gen(wlev) p(0.01)

winsor ppe,gen(wppe) p(0.01)

winsor tobin,gen(wtobin) p(0.01)

winsor age,gen(wage) p(0.01)

winsor mshare,gen(wmshare) p(0.01)

winsor indp,gen(windp) p(0.01)

winsor board,gen(wboard) p(0.01)

winsor lnfirm,gen(wlnfirm) p(0.01)

***********************

***** Baseline regression

gen fsts_fs1=fsts*fs10

gen fsts_fsrate=fsts*fsrate

winsor fsts_fsrate,gen(wfsts_fsrate) p(0.01)

winsor fsts_fs1,gen(wfsts_fs1) p(0.01)

fsum wopm wfsts fs10 wfsrate wsize wlev wppe wtobin wlnfirm wage wmshare windp dual wboard soe,f(10.4)s(n mean sd min p25 median p75 max)

ttest wfsts,by(fs10)

ttest wopm,by(fs10)

eststo: qui reg wopm wfsts wsize wlev wppe wtobin wlnfirm wage wmshare windp dual wboard soe i.fyr i.ind i.place,r

eststo: qui reg wopm wfsts fs10 wfsts_fs1 wsize wlev wppe wtobin wlnfirm wage wmshare windp dual wboard soe,r

eststo: qui reg wopm wfsts fs10 wfsts_fs1 wsize wlev wppe wtobin wlnfirm wage wmshare windp dual wboard soe i.fyr i.ind i.place,r

eststo: qui reg wopm wfsts wfsrate wfsts_fsrate wsize wlev wppe wtobin wlnfirm wage wmshare windp dual wboard soe,r

eststo: qui reg wopm wfsts wfsrate wfsts_fsrate wsize wlev wppe wtobin wlnfirm wage wmshare windp dual wboard soe i.fyr i.ind i.place,r

esttab ,replace nonumbers mtitles r2 ar2 scalars(N N_clust F) star(* 0.1 ** 0.05 *** 0.01) b(%8.3f)

eststo clear

**** Dynamic Adjustment

gen fsts_pre=pre*fsts

gen fsts_fs=fs10*fsts

gen fsts_aft=aft*fsts

winsor fsts_fs,gen(wfsts_fs) p(0.01)

winsor fsts_pre,gen(wfsts_pre) p(0.01)

winsor fsts_aft,gen(wfsts_aft) p(0.01)

eststo: qui reg wopm wfsts pre wfsts_pre wsize wlev wppe wtobin wlnfirm wage wmshare windp dual wboard soe i.fyr i.ind i.place,r

eststo: qui reg wopm wfsts fs10 wfsts_fs wsize wlev wppe wtobin wlnfirm wage wmshare windp dual wboard soe i.fyr i.ind i.place,r

eststo: qui reg wopm wfsts aft wfsts_aft wsize wlev wppe wtobin wlnfirm wage wmshare windp dual wboard soe i.fyr i.ind i.place,r

eststo: qui reg wopm wfsts pre fs10 aft wfsts_pre wfsts_fs wfsts_aft wsize wlev wppe wtobin wlnfirm wage wmshare windp dual wboard soe i.fyr i.ind i.place,r

esttab ,replace nonumbers mtitles r2 ar2 scalars(N N_clust F) star(* 0.1 ** 0.05 *** 0.01) b(%8.3f)

eststo clear

****************

* Robustness test

***1. PSM+OLS

use psmdata,clear

*winsor

winsor opm,gen(wopm) p(0.01)

winsor fsts,gen(wfsts) p(0.01)

winsor fsrate,gen(wfsrate) p(0.01)

winsor size,gen(wsize) p(0.01)

winsor lev,gen(wlev) p(0.01)

winsor ppe,gen(wppe) p(0.01)

winsor tobin,gen(wtobin) p(0.01)

winsor age,gen(wage) p(0.01)

winsor mshare,gen(wmshare) p(0.01)

winsor indp,gen(windp) p(0.01)

winsor board,gen(wboard) p(0.01)

winsor lnfirm,gen(wlnfirm) p(0.01)

*regression

gen fsts_fs1=fsts*fs10

gen fsts_fsrate=fsts*fsrate

winsor fsts_fsrate,gen(wfsts_fsrate) p(0.01)

winsor fsts_fs1,gen(wfsts_fs1) p(0.01)

eststo: qui reg wopm wfsts fs10 wfsts_fs1 wsize wlev wppe wtobin wlnfirm wage wmshare windp dual wboard soe,r

eststo: qui reg wopm wfsts fs10 wfsts_fs1 wsize wlev wppe wtobin wlnfirm wage wmshare windp dual wboard soe i.fyr i.ind i.place,r

eststo: qui reg wopm wfsts wfsrate wfsts_fsrate wsize wlev wppe wtobin wlnfirm wage wmshare windp dual wboard soe,r

eststo: qui reg wopm wfsts wfsrate wfsts_fsrate wsize wlev wppe wtobin wlnfirm wage wmshare windp dual wboard soe i.fyr i.ind i.place,r

esttab ,replace nonumbers mtitles r2 ar2 scalars(N N_clust F) star(* 0.1 ** 0.05 *** 0.01) b(%8.3f)

eststo clear

**2. Heckman two-stage

use data2003-2021,clear

egen miss=rmiss(stkcd fyr opm fsts fs10 size lev ppe dual indp mshare board lnfirm age tobin soe st ind)

tab miss

drop if miss!=0

drop miss

tab fyr

winsor opm,gen(wopm) p(0.01)

winsor fsts,gen(wfsts) p(0.01)

winsor fsrate,gen(wfsrate) p(0.01)

winsor size,gen(wsize) p(0.01)

winsor lev,gen(wlev) p(0.01)

winsor ppe,gen(wppe) p(0.01)

winsor tobin,gen(wtobin) p(0.01)

winsor age,gen(wage) p(0.01)

winsor mshare,gen(wmshare) p(0.01)

winsor indp,gen(windp) p(0.01)

winsor board,gen(wboard) p(0.01)

winsor lnfirm,gen(wlnfirm) p(0.01)

winsor lninvest,gen(wlninvest) p(0.01)

winsor growth,gen(wgrowth) p(0.01)

winsor ldiv,gen(wldiv) p(0.01)

eststo: qui probit fs10 wldiv wlninvest wsize wlev wppe wtobin wlnfirm wage wmshare windp dual wboard soe i.ind i.fyr i.place,r

esttab ,replace nonumbers mtitles r2 ar2 scalars(N N_clust F) star(* 0.1 ** 0.05 *** 0.01) b(%8.3f)

estimate store first

predict y_hat,xb

gen pdf=normalden(y_hat)

gen cdf=normal(y_hat)

gen imr=pdf/cdf

xtset stkcd fyr

gen fsts_fs1=fsts*fs10

gen fsts_fsrate=fsts*fsrate

winsor fsts_fsrate,gen(wfsts_fsrate) p(0.01)

winsor fsts_fs1,gen(wfsts_fs1) p(0.01)

eststo: qui reg wopm wfsts fs10 wfsts_fs1 wsize wlev wppe wtobin wlnfirm wage wmshare windp dual wboard soe imr i.fyr i.ind i.place,r

eststo: qui reg wopm wfsts wfsrate wfsts_fsrate wsize wlev wppe wtobin wlnfirm wage wmshare windp dual wboard soe imr i.fyr i.ind i.place,r

esttab ,replace nonumbers mtitles r2 ar2 scalars(N N_clust F) star(* 0.1 ** 0.05 *** 0.01) b(%8.3f)

eststo clear

*3. Replace explanatory variables

use data2003-2021,clear

egen miss=rmiss(stkcd fyr opm intnpcs fs1 fs15 fs10 fsrate size lev ppe dual indp mshare board lnfirm age tobin soe st ind)

tab miss

drop if miss!=0

drop miss

tab fyr

*winsor

winsor opm,gen(wopm) p(0.01)

winsor fsts,gen(wfsts) p(0.01)

winsor fsrate,gen(wfsrate) p(0.01)

winsor size,gen(wsize) p(0.01)

winsor lev,gen(wlev) p(0.01)

winsor ppe,gen(wppe) p(0.01)

winsor tobin,gen(wtobin) p(0.01)

winsor age,gen(wage) p(0.01)

winsor mshare,gen(wmshare) p(0.01)

winsor indp,gen(windp) p(0.01)

winsor board,gen(wboard) p(0.01)

winsor lnfirm,gen(wlnfirm) p(0.01)

xtset stkcd fyr

gen intnpcs_fs10=intnpcs*fs10

gen intnpcs_fsrate=intnpcs*fsrate

gen fsts_fs1=fsts*fs1

gen fsts_fs15=fsts*fs15

gen intnpcs_fs1=intnpcs*fs1

gen intnpcs_fs15=intnpcs*fs15

winsor intnpcs_fsrate,gen(wintnpcs_fsrate) p(0.01)

winsor fsts_fs1,gen(wfsts_fs1) p(0.01)

winsor fsts_fs15,gen(wfsts_fs15) p(0.01)

eststo: qui reg wopm intnpcs fs10 intnpcs_fs10 wsize wlev wppe wtobin wlnfirm wage wmshare windp dual wboard soe i.fyr i.ind i.place,r

eststo: qui reg wopm intnpcs wfsrate wintnpcs_fsrate wsize wlev wppe wtobin wlnfirm wage wmshare windp dual wboard soe i.fyr i.ind i.place,r

eststo: qui reg wopm wfsts fs1 wfsts_fs1 wsize wlev wppe wtobin wlnfirm wage wmshare windp dual wboard soe i.fyr i.ind i.place,r

eststo: qui reg wopm wfsts fs15 wfsts_fs15 wsize wlev wppe wtobin wlnfirm wage wmshare windp dual wboard soe i.fyr i.ind i.place,r

eststo: qui reg wopm intnpcs fs1 intnpcs_fs1 wsize wlev wppe wtobin wlnfirm wage wmshare windp dual wboard soe i.fyr i.ind i.place,r

eststo: qui reg wopm intnpcs fs15 intnpcs_fs15 wsize wlev wppe wtobin wlnfirm wage wmshare windp dual wboard soe i.fyr i.ind i.place,r

esttab ,replace nonumbers mtitles r2 ar2 scalars(N N_clust F) star(* 0.1 ** 0.05 *** 0.01) b(%8.3f)

eststo clear

**4. Sample Adjustment

use data2003-2021,clear

tab fyr

drop if fyr<=2006

egen miss=rmiss(stkcd fyr opm fsts fs10 fsrate size lev ppe dual indp mshare board lnfirm age tobin soe st ind)

tab miss

drop if miss!=0

drop miss

tab fyr

*winsor

winsor opm,gen(wopm) p(0.01)

winsor fsts,gen(wfsts) p(0.01)

winsor fsrate,gen(wfsrate) p(0.01)

winsor size,gen(wsize) p(0.01)

winsor lev,gen(wlev) p(0.01)

winsor ppe,gen(wppe) p(0.01)

winsor tobin,gen(wtobin) p(0.01)

winsor age,gen(wage) p(0.01)

winsor mshare,gen(wmshare) p(0.01)

winsor indp,gen(windp) p(0.01)

winsor board,gen(wboard) p(0.01)

winsor lnfirm,gen(wlnfirm) p(0.01)

xtset stkcd fyr

gen fsts_fs1=fsts*fs10

gen fsts_fsrate=fsts*fsrate

winsor fsts_fsrate,gen(wfsts_fsrate) p(0.01)

winsor fsts_fs1,gen(wfsts_fs1) p(0.01)

eststo: qui reg wopm wfsts fs10 wfsts_fs1 wsize wlev wppe wtobin wlnfirm wage wmshare windp dual wboard soe,r

eststo: qui reg wopm wfsts fs10 wfsts_fs1 wsize wlev wppe wtobin wlnfirm wage wmshare windp dual wboard soe i.fyr i.ind i.place,r

eststo: qui reg wopm wfsts wfsrate wfsts_fsrate wsize wlev wppe wtobin wlnfirm wage wmshare windp dual wboard soe,r

eststo: qui reg wopm wfsts wfsrate wfsts_fsrate wsize wlev wppe wtobin wlnfirm wage wmshare windp dual wboard soe i.fyr i.ind i.place,r

esttab ,replace nonumbers mtitles r2 ar2 scalars(N N_clust F) star(* 0.1 ** 0.05 *** 0.01) b(%8.3f)

eststo clear

*****************

*** Mechanism analysis

use data2003-2021,clear

egen miss=rmiss(stkcd fyr opm fsts fs10 fsrate tfp_lp sfr cor size lev ppe dual indp mshare board lnfirm age tobin soe st ind)

tab miss

drop if miss!=0

drop miss

tab fyr

*winsor

winsor opm,gen(wopm) p(0.01)

winsor fsts,gen(wfsts) p(0.01)

winsor fsrate,gen(wfsrate) p(0.01)

winsor size,gen(wsize) p(0.01)

winsor lev,gen(wlev) p(0.01)

winsor ppe,gen(wppe) p(0.01)

winsor tobin,gen(wtobin) p(0.01)

winsor age,gen(wage) p(0.01)

winsor mshare,gen(wmshare) p(0.01)

winsor indp,gen(windp) p(0.01)

winsor board,gen(wboard) p(0.01)

winsor lnfirm,gen(wlnfirm) p(0.01)

winsor cor,gen(wcor) p(0.01)

winsor sfr,gen(wsfr) p(0.01)

winsor tfp_lp,gen(wtfp_lp) p(0.01)

xtset stkcd fyr

eststo: qui reg wtfp_lp fs10 wsize wlev wppe wtobin wmshare windp dual wboard soe i.fyr i.ind i.place if fsts>0,r

eststo: qui reg wtfp_lp wfsrate wsize wlev wppe wtobin wmshare windp dual wboard soe i.fyr i.ind i.place if fsts>0,r

eststo: qui reg wcor fs10 wsize wlev wppe wtobin wmshare windp dual wboard soe i.fyr i.ind i.place if fsts>0,r

eststo: qui reg wcor wfsrate wsize wlev wppe wtobin wmshare windp dual wboard soe i.fyr i.ind i.place if fsts>0,r

eststo: qui reg wsfr fs10 wsize wlev wppe wtobin wmshare windp dual wboard soe i.fyr i.ind i.place if fsts>0,r

eststo: qui reg wsfr wfsrate wsize wlev wppe wtobin wmshare windp dual wboard soe i.fyr i.ind i.place if fsts>0,r

esttab ,replace nonumbers mtitles r2 ar2 scalars(N N_clust F) star(* 0.1 ** 0.05 *** 0.01) b(%8.3f)

eststo clear

**********

*** Heterogeneity analysis among foreign shareholders

use data2003-2021,clear

egen miss=rmiss(stkcd fyr opm fsts lon fmult size lev ppe dual indp mshare board lnfirm age tobin soe st ind)

tab miss

drop if miss!=0

drop miss

tab fyr

*winsor

winsor opm,gen(wopm) p(0.01)

winsor fsts,gen(wfsts) p(0.01)

winsor fsrate,gen(wfsrate) p(0.01)

winsor size,gen(wsize) p(0.01)

winsor lev,gen(wlev) p(0.01)

winsor ppe,gen(wppe) p(0.01)

winsor tobin,gen(wtobin) p(0.01)

winsor age,gen(wage) p(0.01)

winsor mshare,gen(wmshare) p(0.01)

winsor indp,gen(windp) p(0.01)

winsor board,gen(wboard) p(0.01)

winsor lnfirm,gen(wlnfirm) p(0.01)

winsor lon,gen(wlon) p(0.01)

xtset stkcd fyr

gen fsts_lon=fsts*lon

gen fsts_fmult=fsts*fmult

winsor fsts_lon,gen(wfsts_lon) p(0.01)

winsor fsts_fmult,gen(wfsts_fmult) p(0.01)

eststo: qui reg wopm wfsts wlon wfsts_lon wsize wlev wppe wtobin wlnfirm wage wmshare windp dual wboard soe ,r

eststo: qui reg wopm wfsts wlon wfsts_lon wsize wlev wppe wtobin wlnfirm wage wmshare windp dual wboard soe i.fyr i.ind i.place,r

eststo: qui reg wopm wfsts fmult wfsts_fmult wsize wlev wppe wtobin wlnfirm wage wmshare windp dual wboard soe ,r

eststo: qui reg wopm wfsts fmult wfsts_fmult wsize wlev wppe wtobin wlnfirm wage wmshare windp dual wboard soe i.fyr i.ind i.place,r

esttab ,replace nonumbers mtitles r2 ar2 scalars(N N_clust F) star(* 0.1 ** 0.05 *** 0.01) b(%8.3f)

eststo clear

*****************

*** Heterogeneity analysis among firms

use data2003-2021,clear

egen miss=rmiss(stkcd fyr opm fsts fs10 fsrate otps ofirm pol size lev ppe dual indp cintd mshare board lnfirm age tobin soe market nonstate law st ind)

tab miss

drop if miss!=0

drop miss

tab fyr

*winsor

winsor opm,gen(wopm) p(0.01)

winsor fsts,gen(wfsts) p(0.01)

winsor fsrate,gen(wfsrate) p(0.01)

winsor size,gen(wsize) p(0.01)

winsor lev,gen(wlev) p(0.01)

winsor ppe,gen(wppe) p(0.01)

winsor tobin,gen(wtobin) p(0.01)

winsor age,gen(wage) p(0.01)

winsor mshare,gen(wmshare) p(0.01)

winsor indp,gen(windp) p(0.01)

winsor board,gen(wboard) p(0.01)

winsor lnfirm,gen(wlnfirm) p(0.01)

xtset stkcd fyr

gen fsts_fs1=fsts*fs10

gen fsts_fsrate=fsts*fsrate

winsor fsts_fsrate,gen(wfsts_fsrate) p(0.01)

winsor fsts_fs1,gen(wfsts_fs1) p(0.01)

*****soe

*eststo: qui reg wopm wfsts fs10 wfsts_fs1 wsize wlev wppe wtobin wlnfirm wage wmshare windp dual wboard soe i.fyr i.ind i.place if soe==1,r

eststo: qui reg wopm wfsts wfsrate wfsts_fsrate wsize wlev wppe wtobin wlnfirm wage wmshare windp dual wboard soe i.fyr i.ind i.place if soe==1,r

*eststo: qui reg wopm wfsts fs10 wfsts_fs1 wsize wlev wppe wtobin wlnfirm wage wmshare windp dual wboard soe i.fyr i.ind i.place if soe==0,r

eststo: qui reg wopm wfsts wfsrate wfsts_fsrate wsize wlev wppe wtobin wlnfirm wage wmshare windp dual wboard soe i.fyr i.ind i.place if soe==0,r

eststo: qui reg wopm wfsts wfsrate wfsts_fsrate wsize wlev wppe wtobin wlnfirm wage wmshare windp dual wboard soe i.fyr i.ind i.place if fc2==0,r

eststo: qui reg wopm wfsts wfsrate wfsts_fsrate wsize wlev wppe wtobin wlnfirm wage wmshare windp dual wboard soe i.fyr i.ind i.place if fc2==2,r

esttab ,replace nonumbers mtitles r2 ar2 scalars(N N_clust F) star(* 0.1 ** 0.05 *** 0.01) b(%8.3f)

eststo clear

**institution

bysort fyr ind:egen mmarket=mean(market)

gen mardum=(market>mmarket)

bysort fyr ind:egen mnonstate=mean(nonstate)

gen nondum=(nonstate>mnonstate)

bysort fyr ind:egen mlaw=mean(law)

gen lawdum=(law>mlaw)

*market index

*eststo: qui reg wopm wfsts fs10 wfsts_fs1 wsize wlev wppe wtobin wlnfirm wage wmshare windp dual wboard soe i.fyr i.ind i.place if mardum==0,r

*eststo: qui reg wopm wfsts fs10 wfsts_fs1 wsize wlev wppe wtobin wlnfirm wage wmshare windp dual wboard soe i.fyr i.ind i.place if mardum==1,r

*eststo: qui reg wopm wfsts fs10 wfsts_fs1 wsize wlev wppe wtobin wlnfirm wage wmshare windp dual wboard soe i.fyr i.ind i.place if nondum==0,r

*eststo: qui reg wopm wfsts fs10 wfsts_fs1 wsize wlev wppe wtobin wlnfirm wage wmshare windp dual wboard soe i.fyr i.ind i.place if nondum==1,r

*eststo: qui reg wopm wfsts fs10 wfsts_fs1 wsize wlev wppe wtobin wlnfirm wage wmshare windp dual wboard soe i.fyr i.ind i.place if lawdum==0,r

*eststo: qui reg wopm wfsts fs10 wfsts_fs1 wsize wlev wppe wtobin wlnfirm wage wmshare windp dual wboard soe i.fyr i.ind i.place if lawdum==1,r

eststo: qui reg wopm wfsts wfsrate wfsts_fsrate wsize wlev wppe wtobin wlnfirm wage wmshare windp dual wboard soe i.fyr i.ind i.place if mardum==0,r

eststo: qui reg wopm wfsts wfsrate wfsts_fsrate wsize wlev wppe wtobin wlnfirm wage wmshare windp dual wboard soe i.fyr i.ind i.place if mardum==1,r

eststo: qui reg wopm wfsts wfsrate wfsts_fsrate wsize wlev wppe wtobin wlnfirm wage wmshare windp dual wboard soe i.fyr i.ind i.place if nondum==0,r

eststo: qui reg wopm wfsts wfsrate wfsts_fsrate wsize wlev wppe wtobin wlnfirm wage wmshare windp dual wboard soe i.fyr i.ind i.place if nondum==1,r

eststo: qui reg wopm wfsts wfsrate wfsts_fsrate wsize wlev wppe wtobin wlnfirm wage wmshare windp dual wboard soe i.fyr i.ind i.place if lawdum==0,r

eststo: qui reg wopm wfsts wfsrate wfsts_fsrate wsize wlev wppe wtobin wlnfirm wage wmshare windp dual wboard soe i.fyr i.ind i.place if lawdum==1,r

esttab ,replace nonumbers mtitles r2 ar2 scalars(N N_clust F) star(* 0.1 ** 0.05 *** 0.01) b(%8.3f)

eststo clear

*overseas firm

bysort fyr ind:egen mofirm=mean(ofirm)

gen otpsd=(otps>0)

gen ofirmd=(ofirm>mofirm)

*eststo: qui reg wopm wfsts fs10 wfsts_fs1 wsize wlev wppe wtobin wlnfirm wage wmshare windp dual wboard soe i.fyr i.ind i.place if otpsd==0,r

eststo: qui reg wopm wfsts wfsrate wfsts_fsrate wsize wlev wppe wtobin wlnfirm wage wmshare windp dual wboard soe i.fyr i.ind i.place if otpsd==0,r

*eststo: qui reg wopm wfsts fs10 wfsts_fs1 wsize wlev wppe wtobin wlnfirm wage wmshare windp dual wboard soe i.fyr i.ind i.place if otpsd==1,r

eststo: qui reg wopm wfsts wfsrate wfsts_fsrate wsize wlev wppe wtobin wlnfirm wage wmshare windp dual wboard soe i.fyr i.ind i.place if otpsd==1,r

*eststo: qui reg wopm wfsts fs10 wfsts_fs1 wsize wlev wppe wtobin wlnfirm wage wmshare windp dual wboard soe i.fyr i.ind i.place if ofirmd==0,r

eststo: qui reg wopm wfsts wfsrate wfsts_fsrate wsize wlev wppe wtobin wlnfirm wage wmshare windp dual wboard soe i.fyr i.ind i.place if ofirmd==0,r

*eststo: qui reg wopm wfsts fs10 wfsts_fs1 wsize wlev wppe wtobin wlnfirm wage wmshare windp dual wboard soe i.fyr i.ind i.place if ofirmd==1,r

eststo: qui reg wopm wfsts wfsrate wfsts_fsrate wsize wlev wppe wtobin wlnfirm wage wmshare windp dual wboard soe i.fyr i.ind i.place if ofirmd==1,r

esttab ,replace nonumbers mtitles r2 ar2 scalars(N N_clust F) star(* 0.1 ** 0.05 *** 0.01) b(%8.3f)

eststo clear

*industry

*eststo: qui xtreg wopm wfsts fs10 wfsts_fs1 wsize wlev wppe wtobin wlnfirm wage wmshare windp dual wboard soe i.fyr i.ind i.place if poll==0,r

eststo: qui xtreg wopm wfsts wfsrate wfsts_fsrate wsize wlev wppe wtobin wlnfirm wage wmshare windp dual wboard soe i.fyr i.ind i.place if poll==0,r

*eststo: qui xtreg wopm wfsts fs10 wfsts_fs1 wsize wlev wppe wtobin wlnfirm wage wmshare windp dual wboard soe i.fyr i.ind i.place if poll==1,r

eststo: qui xtreg wopm wfsts wfsrate wfsts_fsrate wsize wlev wppe wtobin wlnfirm wage wmshare windp dual wboard soe i.fyr i.ind i.place if poll==1,r

*eststo: qui reg wopm wfsts fs10 wfsts_fs1 wsize wlev wppe wtobin wlnfirm wage wmshare windp dual wboard soe i.fyr i.ind i.place if cintd==0,r

eststo: qui reg wopm wfsts wfsrate wfsts_fsrate wsize wlev wppe wtobin wlnfirm wage wmshare windp dual wboard soe i.fyr i.ind i.place if cintd==0,r

*eststo: qui reg wopm wfsts fs10 wfsts_fs1 wsize wlev wppe wtobin wlnfirm wage wmshare windp dual wboard soe i.fyr i.ind i.place if cintd==1,r

eststo: qui reg wopm wfsts wfsrate wfsts_fsrate wsize wlev wppe wtobin wlnfirm wage wmshare windp dual wboard soe i.fyr i.ind i.place if cintd==1,r

esttab ,replace nonumbers mtitles r2 ar2 scalars(r2_w r2_bb N N_clust F) star(* 0.1 ** 0.05 *** 0.01) b(%8.3f)

eststo clear
